# An Unrevealed Molecular Function of Corannulene Buckybowl Glycoconjugates in Selective Tumor Annihilation by Targeting the Cancer‐Specific Warburg Effect

**DOI:** 10.1002/advs.202105315

**Published:** 2022-03-07

**Authors:** Shengnan Liu, Ziru Sun, Min Liang, Weijie Song, Ru Zhang, Yunli Shi, Yujun Cui, Qingzhi Gao

**Affiliations:** ^1^ Institute of Molecular Plus Frontiers Science Center for Synthetic Biology (Ministry of Education of China) Tianjin Key Laboratory for Modern Drug Delivery and High‐Efficiency Tianjin University 92 Weijin Road Nankai District Tianjin 300072 P. R. China; ^2^ Transplantation Center Tianjin First Central Hospital 24 Fukang Road Nankai District Tianjin 300192 P. R. China; ^3^ Central Institute of Pharmaceutical Research CSPC Pharmaceutical Group 226 Huanhe Road Shijiazhuang Hebei 050035 P. R. China; ^4^ Tianjin Medical University Cancer Institute and Hospital National Clinical Research Center for Cancer West Huanhu Road Hexi District Tianjin 300060 P. R. China; ^5^ Department of Biology Gudui BioPharma Technology Inc. Huayuan Industrial Park 5 Lanyuan Road Tianjin 300384 P. R. China

**Keywords:** anticancer, corannulene, DNA binding, glucose transporter, Warburg effect

## Abstract

The biomedical application of corannulene *π*‐bowls is historically limited by low solubility and bioavailability despite the potential in their unique electronic properties for new functional materials. Herein, the unexpected role and molecular mechanism of Corranulene *π*‐bowls are uncovered in biomedical applications as an effective anticancer agent for Warburg effect mediated selective tumor targeting. The corannulene triazolyl monosaccharides Cor‐sugars exhibit highly potent cytotoxicity against human cancer cells and effectively inhibit xenograft growth of hyperglycolytic tumors. Particularly, the galactose‐conjugated Cor‐gal exhibits superior in vivo anticancer efficacy in A549 tumor models with outstanding safety profile compared to doxorubicin. Moreover, the combined treatment of Cor‐gal with immune checkpoint inhibitor results in an effective synergy in treating H460 human lung carcinoma. An uptake mechanism study reveals that Cor‐sugars exploit tumor‐specific glucose transporter glucose transporter 1 (GLUT1) for targeted cell delivery and intra‐tumoral accumulation through the cancer‐specific Warburg effect. Their significant anticancer activity is attributed to multiphasic DNA‐binding and cell cycle alteration effects. This study uncovers new molecular properties of corannulene buckybowl and enabling their potential new applications in biomedical engineering.

## Graphical abstract

Sugar‐conjugated corannulene *π*‐bowls possess a new molecular function in DNA interactive anticancer therapy. Corannulene‐sugar conjugates can be transported through glucose transporter 1 (GLUT1) for cancer cell uptake and effectively annihilate xenograft tumors by leveraging the Warburg effect. The anticancer activity relies on a unique multiphasic DNA binding mechanism of the curved corannulene molecule and selectively induces cell cycle arrest in tumor cells.

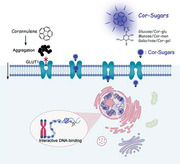

## 1 Introduction

Since corannulene was synthesized by Lawton and Barth from a multistep process in 1966,^[^ ^1^ ^]^ it has become one of the most important molecular tool in material science and nanotechnological researches.^[^ ^2^, ^3^ ^]^ However, the challenges and difficulties involved in the synthetic method precluded the systematic investigation of this *π*‐bowl molecule until a simpler preparation was developed by Scott and his co‐workers in early 1990s,^[^ ^4^, ^5^ ^]^ In 2012, Siegel’ s group discovered a kilogram‐scale synthesis of corannulene,^[^ ^6^ ^]^ and accordingly, a variety of corannulene derivatives have been prepared and therefore available for studies in different disciplinary fields.^[^ ^7^, ^8^, ^9^, ^10^, ^11^ ^]^ While a large number of corannulene‐based analogs have been reported, due to the extremely low solubility of most of the compounds under physiological conditions, there are very few examples that focus on the importance of this *π*‐bowl molecule in pharmaceutical applications. The most relevant literature, to the best of our knowledge, reporting the biological behavior of unaggregated corannulene derivative is the in vitro study of a pentavalent ganglioside of corannulene on cholera toxin inhibition.^[^ ^12^ ^]^ Therefore, very little is known about the molecular‐based profiles and potential pharmacological properties of corannulene *π*‐bowl in medicinal and biopharmaceutical chemistry.

In this work, we report the first survey of corranulene monoglycoconjugates (Cor‐sugars) as a novel class of anticancer agents with their synthesis, cytotoxicity, in vivo efficacy, molecular uptake and their unique tumor targeting mechanisms. The rationale of the design concept for conjugating monosaccharides through triazolyl moiety with corannulene was driven by the following objectives: 1) improving water solubility through glycoconjugation to demonstrate the physiological and pharmacological properties associated with the unaggregated single molecule, 2) enhancing the fluorogenic property of corannulene by conjugation of triazolyl *π*‐system to enable their luminescence‐based in vitro and in vivo self‐probing, 3) exploring the DNA‐interacting potential of the extensively conjugated *π*‐system of Cor‐sugars in cancer therapy, and 4) leveraging the high expression of glucose transporters (GLUTs) in cancer cells (the Warburg effect) for selective tumor uptake.

With the rationally designed Cor‐sugars, our study results revealed that the corannulene triazolyl monosaccharides can act as potent DNA damaging agents with preferential cytotoxicity against a panel of human cancer cells but spares normal cells. In in vivo, the galactose conjugated corannulene compound: Cor‐gal effectively suppressed the growth of A549 lung cancer in tumor‐bearing mice by targeting GLUT1. Furthermore, Cor‐gal resulted in a near complete eradication of H460 lung carcinoma in xenograft mice when used in combination with immune checkpoint inhibitor (CPI) Atezolizumab. This study provides the first evidence that the corannulene *π*‐bowl derivatives possess a great potential in selective tumor targeting and presents preliminary insights into the molecular behavior of the curved buckybowls on DNA interaction in biological systems.

## 2 Results

### 2.1 Synthesis and Fluorescent Characteristics of the Cor‐Sugars

**Figure 1:**
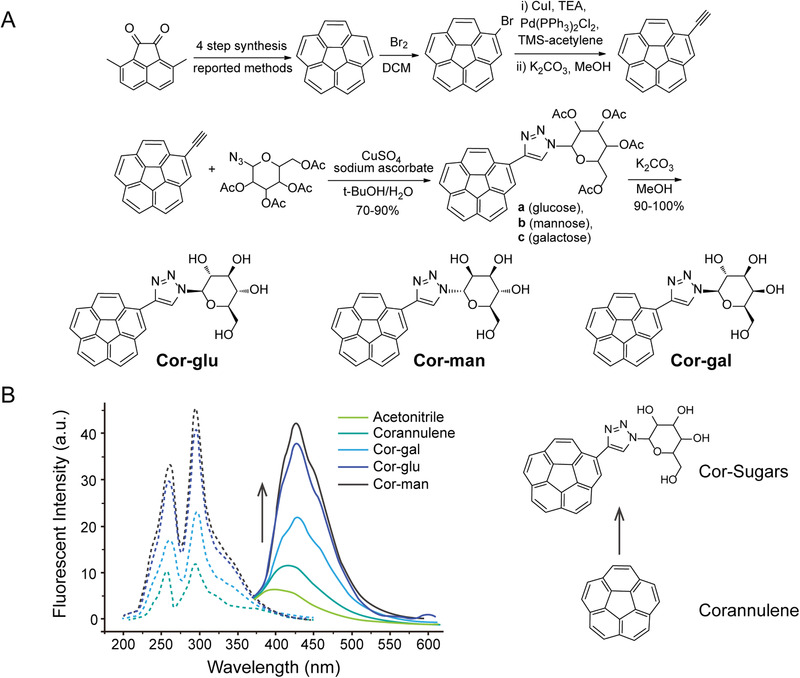
Synthetic method, excitation and emission spectra of Cor‐sugars. A) Preparation of the corannulene intermediates and the corannulene trizolyl sugar conjugates (Cor‐sugars). B) The maximum fluorescence excitation and emission spectrum of Cor‐sugars were recorded at *λ*_ex_ = 300 nm and *λ*_em_ = 420 nm, respectively, with a significant redshift and hyperchromic effect relative to corannulene.

The synthesis of the corranulene triazolyl monosaccharides were carried out in five steps with an overall yield of up to 46%. Briefly, preparations for corannulene,^[^ ^13^ ^]^ corannulene bromide,^[^ ^14^ ^]^ and ethynylcorannulene^[^ ^15^ ^]^ were followed the reported methods. As a new finding in the current study, unlike the thermal Huisgen 1,3‐dipolar cycloaddition,^[^ ^16^ ^]^ the copper‐catalyzed click chemistry of ethynylcorannulene with sugar azides allows the selective production of the 1,4‐disubstituted regioisomers in a very high yield (**Figure** **1**A). In addition, the anomeric stereochemistry of the sugar appendants in each Cor‐sugars was determined to be a pure single isomer by 1H NMR analysis of the C1‐proton in the hexose moiety. Namely, Cor‐glu and Cor‐gal were assigned to be *β*‐anomer and Cor‐gal was assigned to be *α*‐anomer.^[^ ^17^, ^18^ ^]^ All new compounds were unambiguously characterized by 1H and 13C NMR, UV, fourier transform infrared (FT‐IR) and mass spectrometry. The purity of each conjugate was confirmed to be >96% by analytical high‐performance liquid chromatography (HPLC) (Figures S1–S15, Supporting Information)

The direct conjugation of the triazolyl group to the corannulene *π*‐system effectively contributes to the delocalization of molecular orbitals which in turn changes the fluorescent properties of the molecule. This change is evidenced by the redshift and hyperchromic effect on both the excitation and emission spectra of the conjugates in comparison with corannulene (Figure 1B and Figure S16–S19, Supporting Information). The enhanced emission property makes the molecules suitable for bioluminescence self‐imaging in vitro and in vivo. In addition, sugar conjugation greatly enhances the molecular polarity and water solubility of the compounds, thus, they can be easily dissolved in physiological buffers, allowing them to serve as potential bioactive substances for reliable and valid physiological investigations (**Table** **1**).^[^ ^19^ ^]^

**Table 1:** Photophysical characterization of the Cor‐sugar compounds in aqueous solution

	Cor‐glu	Cor‐man	Cor‐gal
*λ*_ex_^max^ [nm]^a)^	297	297	297
*λ*_em_^max^ [nm]	451	451	451
*Φ*_f_	14.62%	12.91%	16.65%
*τ*_f_ [ns]	10.12	9.60	8.20
*χ* ^2^	1.12	1.29	1.06

^a)^ *λ*_ex_^max^: fluorescence excitation maximum; *λ*_em_^max^: fluorescence emission maximum; *Φ*_f_: fluorescence quantum yield; *τ*_f_: fluorescence lifetime; *χ* ^2^: chi squared for the fitting of the emission decay.

### 2.2 Cor‐Sugars Exert Potent Anticancer Activity against a Panel of Human Cancer Cells

**Figure 2:**
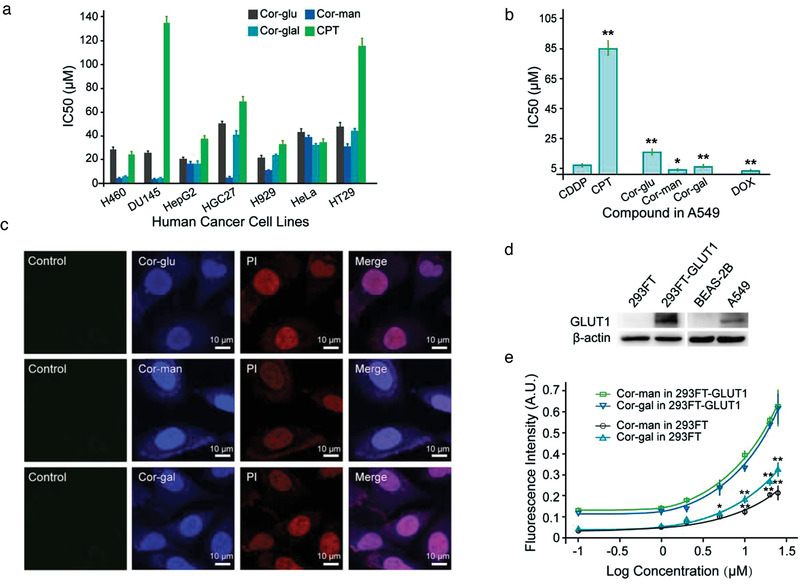
In vitro anticancer activity and GLUT1 dependent cell uptake of Cor‐sugars. a) MTT assay results of Cor‐glu, Cor‐gal, and Cor‐gal against a panel of human cancer cells of different origin. b) Anticancer activity of Cor‐sugars in comparison with cisplatin (CDDP), carboplatin (CPT), and DOX against A549 nonsmall cell lung cancer cells. All experiments were performed in a 72 h assay. **P < 0.01, *P < 0.05 compared with the CDDP treatment group. c) Confocal fluorescence microscopy imaging of the intracellular and nucleus accumulation of Cor‐sugars in A549 cells self‐probed by luminescence of the compounds. 50 × 10^−9^ m of Cor‐sugars for 30 min treatment. d) Western blotting analysis of GLUT1 expression in HEK‐293FT, 293FT‐GLUT1, human normal bronchial epithelial BEAS‐2B, and human nonsmall‐cell lung cancer A549 cells. e) GLUT1‐mediated cellular uptake of Cor‐gal and Cor‐man in different cell lines self‐probed by fluorescence intensity of the test compounds. Untreated 293FT‐GLUT1 and 293FT groups were used as negative control, and the fluorescence intensity of the control groups was subtracted from measurements. Data represent the mean ± standard deviation (SD) of at least three replicates. **P < 0.01, *P < 0.05 for 293FT‐GLUT1 versus corresponding 293FT groups.

We first evaluated the cytotoxicity of Cor‐sugars using the MTT (3‐(4,5‐dimethylthiazol‐2‐yl)‐2,5‐diphenyltetrazolium bromide) assay against a panel of human cancer cell lines, namely, lung (H460), prostate (DU145), liver (HepG2), gastric (human gastric cancer, HGC27), multiple myeloma (H929), cervical (Hela), and colon (HT29) cancers. Surprisingly, all Cor‐sugars effectively halt cancer cell growth with a broad spectrum and exhibited more potent anticancer activity than the platinum‐based DNA‐binding agent carboplatin (**Figure** **2**a and Table S1, Supporting Information). Among the tested cancer cells, the human nonsmall‐cell lung cancer H460 and the androgen receptor positive and hormone‐insensitive human prostate cancer DU145 exhibited very high sensitivity to the mannose‐ and galactose‐derived molecules: Cor‐man and Cor‐gal. Meanwhile, the human gastric carcinoma HGC27 cells were selectively vulnerable to Cor‐gal compared to other two conjugates. We applied the normal lung bronchial epithelial cells (BEAS‐2B) as positive control, and no cytotoxicity effect was observed from treatment with the highest concentration of Cor‐sugars (Table S1, Supporting Information). To further confirm the therapeutic potential of the compounds, we compared the Cor‐sugars with another representative DNA‐intercalating drug, doxorubicin (DOX) as well as the traditional DNA‐alkylating drug cisplatin (CDDP) and carboplatin (CPT) using chemoresistant nonsmall lung cancer A549 cells. As shown in Figure 2b (Table S2, Supporting Information), all Cor‐sugars possessed similar high potencies with IC_50_ values comparable to that of DOX and CDDP, and were much more effective than CPT. Among these compounds, Cor‐gal and Cor‐man were more cytotoxic than Cor‐glu, and both compounds have shown comparable or superior cytotoxicity to CDDP and DOX. This result is in consistent with that observed from the malignant phenotype of H460 lung cancer cell line. We therefore take A549 as model cell line to further investigate the impact of anticancer treatment with Cor‐sugars. Choose A549 for further verification because: 1) our previous study demonstrated that glucose transporter 1 (GLUT1) expression was significantly higher in A549 cells which is suitable for assessing the potential impact of Cor‐sugars by leveraging the tumor Warburg effect^[^ ^20^, ^21^, ^22^ ^]^ and 2) A549 cells and its xenograft have been well characterized as a valuable model in rodent and zebrafish as well for evaluation not only of the tumor regression but also angiogenesis and metastases which allow us to survey in more details the mechanistic behavior of Cor‐sugars.^[^ ^23^, ^24^, ^25^ ^]^

### 2.3 Cor‐Sugars Exploit GLUT1 for Selective Cell Uptake

Based on the intrinsically improved fluorescence emission property of the Cor‐sugars, the cellular uptake of the molecule could be easily assessed with confocal laser scanning microscopy. As shown in Figure 2c, Cor‐sugars showed significant cytoplasmic accumulation in A549 cells which has been confirmed to be expressing high levels of GLUT1 transporters that is upregulated by the Warburg effect mediated tumor glycolysis (Figure 2d).^[^ ^21^, ^22^ ^]^ In addition to the A549 cancer cells, the GLUT1‐dependent intracellular accumulation was further investigated using GLUT1‐transfected human embryonic kidney (HEK) 293FT cells, and by performing a competitive uptake‐inhibition study of Cor‐gal and Cor‐man with a GLUT inhibitor. As the results, significantly enhanced drug uptake in 293FT‐GLUT1 cells was observed compared to the mock‐infected 293FT cells (Figure 2e). In GLUT1 overexpress A549 cells, the cellular uptake of both Cor‐gal and Cor‐man was effectively inhibited by cotreatment of 100 × 10^−6^ m of GLUT inhibitor phloretin (Figure S20, Supporting Information). Phloretin has been shown to be a noncompetitive glucose transport inhibitor with IC_50_ values of 49.0 × 10^−6^, 9.5 × 10^−6^, and 10.0 × 10^−6^ m, respectively for human GLUT1, GLUT3, and GLUT4. These GLUTs are demonstrated to play an important role in cancer hyperglycolysis and sugar uptake leveraged by the Warburg effect.^[^ ^26^, ^27^, ^28^ ^]^ Both results from GLUT1 transfected cell uptake and GLUT inhibitor mediated competitive accumulation assay support the mechanism of cellular transport and intracellular accumulation of Cor‐sugars is regulated at least by the tumor specific transporter GLUT1.

### 2.4 Cor‐Gal Participates Molecular Interaction with Double‐Strand DNA

**Figure 3:**
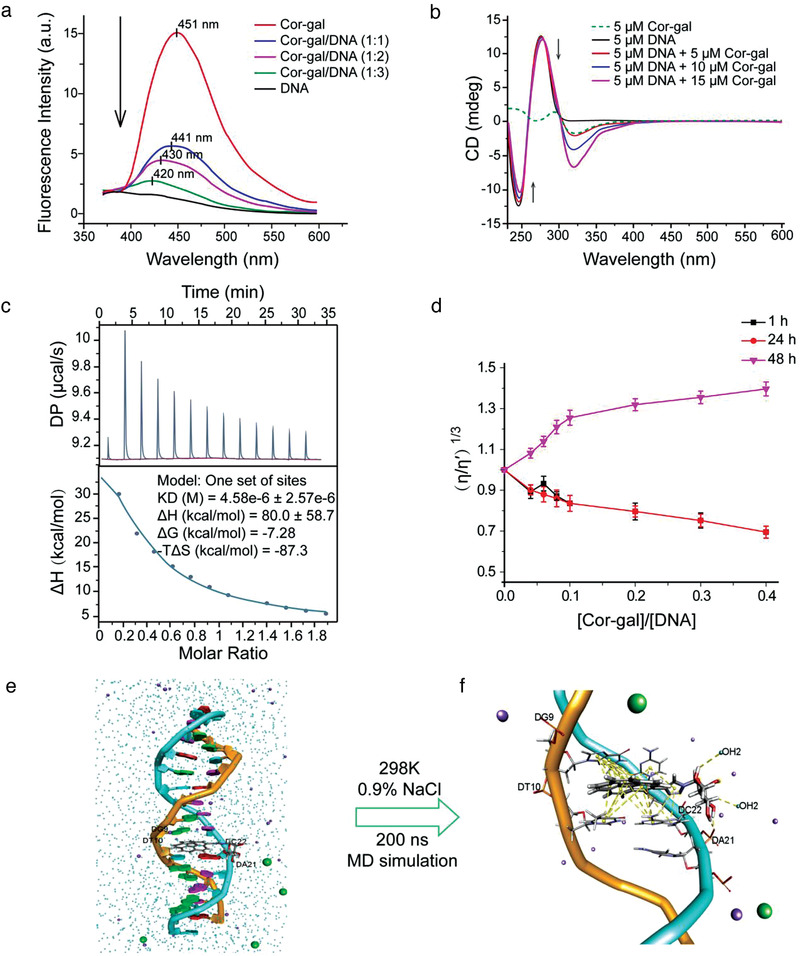
DNA interaction properties of Cor‐sugars. a) Fluorescence quenching of Cor‐gal in the presence of increasing amount of hsDNA. b) CD spectra of hsDNA titrated by increasing amount of Cor‐gal in Tris‐HCl buffer (pH 7.2) at different molar ratio at 25 °C. c) Thermodynamic parameters for instant binding of Cor‐gal with hsDNA by ITC study (10 × 10^−6^ m Cor‐gal were titrated by 100 µM of hsDNA). d) Effects of increasing amounts of Cor‐gal on the relative viscosity of hsDNA at 25 ± 0.1 °C. e) yet another scientific artificial reality application (YASARA) Micro mediated 200 ns molecular dynamics simulation result of Cor‐gal with 15‐mer DNA (PDB: 2MG8) in a 0.9% NaCl solution at 298 K. f) Binding mode of intercalated Cor‐gal in the DNA base pairs after 200 ns MD simulation.

From the self‐probed confocal bioimaging results of cellular uptake of the Cor‐sugars, we have detected a high level of drug accumulation in cell nuclei (Figure 2c). Compared to the nucleus, distribution of the drug molecule in other organelles such as mitochondria and endoplasmic reticulum remained much less abundant (Figure S21, Supporting Information). This observation prompted us to investigate the DNA interaction property of the Cor‐sugars for direct evidence of their anticancer mechanisms. Recently, Liu and Qiu reported a nonplanar polycyclic aromatic hydrocarbons (PAH) [4]helicenium that show enhanced antitumor capability and selectivity by DNA binding mediated cytotoxicity.^[^ ^29^ ^]^ However, as the smallest PAH buchybowl subunit, corannulene analogs have never been explored for DNA interaction activities. Taking the most cytotoxic Cor‐gal compound, we first investigated the direct DNA interaction activity from co‐incubation of Cor‐gal with the double‐stranded herring sperm DNA (hsDNA). Our study revealed that, compared with free Cor‐gal, the luminescence intensity of Cor‐gal in the drug‐DNA complex drastically decreases accompanied with a blueshift from 451 to 420 nm. The decreased fluorescence intensity (FI) in the presence of DNA point to a consequence of static fluorescence quenching with a nonradiation energy transfer between Cor‐gal and the DNA molecule (**Figure** **3**a). In the second, circular dichroism (CD) spectrum of Cor‐sugar/DNA mixture revealed a significant hypochromic shift (up to 35%) at negative band (245 nm) and a notable redshift (>3 nm) of the positive band at 275 nm (Figure 3b and Figures S22 and S23, Supporting Information). This indicates a strong perturbation of the helical structure of the DNA from the ligand binding.

**Figure 4:**
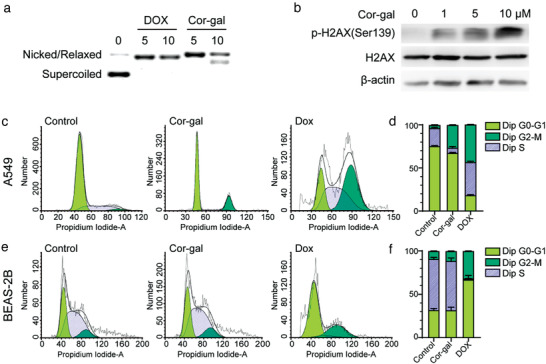
Different cell cycle arrest induction effect between Cor‐gal and DOX in cancer and normal cells. a) Agarose gel electrophoresis of pBR322 plasmid DNA incubated with Cor‐gal (5 × 10^−6^ and 10 × 10^−6^ m Cor‐gal treatment in 5 × 10^−3^ m Tris‐HCl/50 × 10^−3^ m NaCl buffer at 37 °C for 48 h). b) Western blotting and immunocytochemistry for *γ*‐H2AX expression in Cor‐gal treated A549 cells. c) Flow cytometry histograms of Cor‐gal and DOX induced cell cycle arrest in A549 cells. d) Statistical analysis of the percentage of the cell cycle distribution in A549 cells. e) Flow cytometry histograms of Cor‐gal and DOX induced cell cycle arrest in BEAS‐2B normal cells. f) Statistical analysis of the percentage of the cell cycle distribution in drug treated BEAS‐2B normal cells. All data are expressed as the mean ± SD of three independent experiments.

More interestingly, an efficient chirality induction to the bound Cor‐gal has been observed from the CD measurement. As shown in Figure 3b, Cor‐gal exhibits a weak and multimodal CD spectrum in the UV range at room temperature due to the racemization of corannulene moiety through bowl‐to‐bowl inversion.^[^ ^30^, ^31^ ^]^ However, in the DNA complex, a significant increase of the negative Cotton effect curve of corannulene at 320 nm has been recorded indicating that an enantioenriched *π*‐bowl isomer of Cor‐gal has formed during the DNA binding process. To determine the DNA‐binding parameters of the corannulene sugar‐conjugates, the thermodynamic profiles for Cor‐gal with hsDNA have been deciphered utilizing isothermal titration calorimetry (ITC) by a reverse titration method. As shown in **Figure** **4**c, the binding affinity for Cor‐gal was around 4 × 10^−6^ m. Since the standard changes of both Δ*H* and Δ*S* during complex formation have the following contributions: the desolvation change (Δ*H*_des_, Δ*S*_des_), the conformational change of the ligand binding site (Δ*H*_conf_, Δ*S*_conf_), and the enthalpy/entropy changes of formation of the ligand‐DNA interactions (Δ*H*_DNA/ligand_, Δ*S*_DNA/ligand_),^[^ ^32^ ^]^ thus

(1) $$\Delta H=\Delta H_{\mathrm{des}}+\Delta H_{\mathrm{conf}}+\Delta H_{\mathrm{DNA}/\mathrm{Cor}-\mathrm{gal}}$$

(2) $$\Delta S=\Delta S_{\mathrm{des}}+\Delta S_{\mathrm{conf}}+\Delta S_{\mathrm{DNA}/\mathrm{Cor}-\mathrm{gal}}$$

Hence, the ITC results revealed that the instant binding of Cor‐gal is entirely driven by favorable entropy (both positive Δ*H* and *T*Δ*S*) indicating that the binding signature is basically dominated by the desolvation of the Cor‐gal, conformational changes on the DNA‐binding site and the interaction between Cor‐gal and DNA which in total attributed to the hydrophobic nature of Cor‐gal molecule. The ITC results also revealed that Cor‐gal binding can result in higher conformational disorder of DNA as reflected by the increased entropy value.

### 2.5 Cor‐Gal Displays Both Groove and Intercalative Binding with DNA

Since the viscosity of the DNA solution is sensitive to the complex formation of organic drugs or DNA‐binding molecules, we examined the effects of the Cor‐sugars on the specific relative viscosity changes of hsDNA. Interestingly, from the DNA hydrodynamic study, we found that the Cor‐sugar/DNA interaction displays a time‐dependent multiphasic characteristic. Figure 3d and Figure S24 in the Supporting Information summarized the hydrodynamic study results. The values of “relative specific viscosity” (*η/η◦*)^1^ *^/^* ^3^ (*η◦* and *η* are the specific viscosity contributions of DNA in the absence and presence of the Cor‐sugars, respectively) were plotted against the [drug]/[DNA] ratio. The dynamic changes in the relative viscosity of hsDNA induced by the Cor‐sugars with different incubation time revealed multiphasic behavior of the molecular interaction and binding modes between the corannulene *π*‐bowls with DNA. With a short incubation time (1–24 h, Figure 3d and Figure S24, Supporting Information), a large decrease in “relative specific viscosity” was observed, which indicates that the Cor‐sugars cause a decrease in the hydrodynamic length via large‐scale bending or distortion of the DNA structure. Viscosity decrease has often been ascribed to the grooves binding occurs in the process and this phenomenon has been previously documented for various molecules with electrostatic or partial DNA interactions.^[^ ^33^, ^34^, ^35^ ^]^

For the Cor‐sugar compounds, the electrostatic interaction potential can be reasonably explained with the structural characteristics of their increased molecular dipole, hydrogen‐bonding capability of sugar moiety and positively ionizable triazole ring (this is also evidenced by the binding modes studies from molecular dynamics (MD) simulation results, see next section below). With more longer incubation time, as indicated from the 48 h incubation data, our study revealed an efficient increases in the “relative specific viscosity” for Cor‐gal and Cor‐man, which is characteristic of an intercalative binding of the molecules to the DNA. Compared to these two compounds, Cor‐glu reached a plateau at a higher [drug]/[DNA] ratio (>1:10), which is the only indicative of difference for Cor‐glu in DNA binding from other two molecules. To make sure the stability of Cor‐sugars during the long period assay procedures, a stability analysis was performed using Cor‐gal by HPLC method and the sample was confirmed to be stable over 5 d under the assay conditions (Figures S25–S28, Supporting Information).

To anticipate and compare the hypothesized binding modes of Cor‐gal with DNA, we performed a 200 ns molecular dynamics simulation study with the all‐atom AMBER14 force field (200 nanoseconds at 298 K with a constant pressure of 1 atm in phosphate‐buffered saline (PBS) with 0.9% NaCl, pH = 7.4). As template, we adopted a 15‐mer dsDNA structure from the PDB databank with 5’‐(AGGTCACGGTGGCCA):(TGGCCACCGTGACCT)‐3’ sequence (2MG8) and by replacing Cor‐gal with the complexed native ligand. As summarized in Table S3 and Figures S29–S32 in the Supporting Information, the intercalated Cor‐gal are more energetically favorable as the binding energies calculated by mean of Boundary elements approaches for Cor‐gal are significantly more stable than minor‐ and major‐groove bindings (76.5 kJ mol^−1^ vs −122.91 and −161.75 kJ mol^−1^, more positive binding energies indicate more favorable binding of the compound with the DNA in boundary elements theory). On the other hand, between minor and major groove bindings, the more stable binding energy was predicted in minor groove interaction (−122.91 kJ mol^−1^) and the magnitude of DNA bending caused from Cor‐gal binding are obviously more greater than the major groove binding (Figures S30 and S31, Supporting Information, minor groove binding cause more DNA bending). Combine the results from MD analysis and relative viscosity measurement, we can deduce that the minor groove binding might be the more considerable initial binding pattern in DNA solution which slowly change to the intercalative binding in the process of time. The equilibrated Cor‐gal/DNA structure after 200 ns MD simulation process for intercalative binding mode was depicted in Figure 4e,f. In the predicted intercalative binding mode, all types of DNA bases (G‐T‐C‐A) were able to form *π*‐stacking interactions with the corannulene core which make a very stable intercalation complex between the base pairs 5’‐G9/C22‐3’ and 5’‐T10/A21‐3’, respectively.

In conclusion, compared with the planar DNA‐intercalating agents, the Cor‐sugar compounds appear to exhibit multiphasic DNA binding aspect which can be deduced from the fluorescence quenching, instant DNA binding, time dependent relative DNA viscosity changes and the dynamics simulations. The combination of fast electrostatic DNA binding potential plus the slow intercalating effect could be one of the molecular bases for the superior anticancer and safety profiles of the corannulene *π*‐bowls.

### 2.6 Cor‐Gal Promotes DNA Damages in Cancer Cell

The effect of Cor‐gal induced DNA damages were investigated with plasmid DNA cleavage test and DNA damage quantification analysis. Since DNA double‐strand breaks (DSBs) are the most cytotoxic DNA lesions that may cause from a DNA damaging agent, we first conducted a supercoiled (Sc) DNA unwinding study of Cor‐gal by using agarose gel electrophoresis. As shown in Figure 4a, the results shows that Cor‐gal was able to cleave the Sc DNA to nicked or relaxed counterparts as indicated by the formation of the new DNA bands and the disappearance of the parent Sc DNA. It should be noted that a different DNA damage pattern has been detected between Cor‐gal and DOX which currently remains out of our understanding for the mechanistic difference and deserves further investigation. In addition, to further confirm the DNA damage response of cancer cells, we conducted the Western blotting determination of expression changes of histone *γ*‐H2AX (also called p‐H2AX) protein which is indicative of defective DNA repair after DNA damage.^[^ ^36^ ^]^ As indicated in Figure 4b, Cor‐gal dose‐dependently induces increased *γ*‐H2AX expression in A549 cells. This result shows a good correlation with the plasmid DNA cleavage measurements and indicates that Cor‐gal induced DSB accumulation in A549 cells occurs in a drug concentration dependent manner.

### 2.7 Cor‐Gal Induces Cell Cycle Arrest in Cancer Cell but Not in Normal Cells

A cell proliferation survey has been performed by flow cytometry analysis to compare the difference between Cor‐gal and DOX in terms of cell cycle arrest. A549 cancer cells were respectively incubated with 5 × 10^−6^ m of Cor‐gal and DOX for 24 h for cell cycle distribution assessment. Follow the same procedure, BEAS‐2B normal cells were also examined to evaluate the difference in cellular response that may cause from Cor‐gal and for comparison of the two drug molecules.

In A549 cancer cells (Figure 4c,d), Co‐gal significantly arrested cell cycle in G2/M phase (from 3.59% ± 0.15 of blank A549 to 26.34% ± 1.43 Cor‐gal treated, *p *< 0.001) accompanied by a decrease in the proportion of S phase cells (from 20.93% ± 1.30 to 6.08% ± 0.11, *p *< 0.001). However, DOX treated A549 cells undergo different cell cycle modifications with significant increase on both G2/M (from 3.59% ± 0.15 of blank A549 to 43.15% ± 1.76), and S phase cells (from 20.93% ± 1.30 to 38.36% ± 0.40, *p *< 0.001), and accompanied by a remarkable decrease in the proportion of G0‐G1 cells (from 75.48% ± 2.71 to 18.49% ± 1.11, *p *< 0.001). The cell cycle restriction differences between Cor‐gal and DOX in cancer cells, also reflected by the DNA unwinding results of these two molecules in which the different behavior has been identified.

In the noncancerous BEAS‐2B cells, Cor‐gal treatment did not alter the distribution of the cells in almost all cell cycles (Figure 4e,f). By contrast, DOX treated normal cells still demonstrated a significant alteration effect on cell cycle arrest. After DOX treatment, BEAS‐2B cells were arrested mainly in the G1‐G1 phase of the cell cycle (from 31.79% to 66.97%), whereas Cor‐gal causes no changes (31.79% vs 31.74%). In addition to the G0‐G1 cell cycle arrest, drastic decreases of cells in S phase (from 58.13% to 1.74%) accompanied with increment of cells at G2/M phase (from 12.18% to 31.28%) were also observed. These results represent the nonselective toxicity profiles of DOX. Compared to DOX, Cor‐gal did not induce observable cell cycle impairment in BEAS‐2B normal cells (S phase: from 58.13% to 56.07%, G2/M phase: from 10.00% to 12.18%). The differences between Cor‐gal and DOX in the cell cycle alteration effect might be another key aspect for understanding the unexpected role of Cor‐gal in selective tumor targeting.

### 2.8 Cor‐Sugars Exhibit Excellent Safety Profile In Vivo

**Figure 5:**
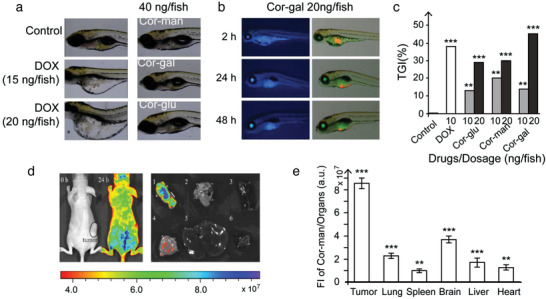
Safety and in vivo antitumor efficacy evaluations in zebrafish and A549 bearing xenograft. Tumor drug accumulation analysis of Cor‐gal in A549‐bearing mice. a) Abnormal phenotypes detected from DOX treatment and no toxicity observed for Cor‐sugars. b) Time‐dependent drug distribution and tumor suppression images in larvae treated with 20 ng per fish Cor‐gal. Left row: Original fluorescence of Cor‐gal (*λ*_ex_ = 295 nm). Right row: Green filter was used without changing the ex. hpi: hours postinjection. c) Inhibition rate of A549 tumor growth after treatment with Cor‐sugars in zebrafish xenograft model. Values are based on the average of the tumor cell fluorescence intensity emitted by the tumor masses (*n* = 30/group) after 48 h treatment. ***P < 0.001, **P < 0.01 compared with the untreated control group. d) Ex vivo tumor accumulation study of Cor‐gal in A549‐bearing mice by fluorescence imaging. e) Quantitative bio‐distribution of Cor‐gal in A549 xenograft mice based on ex vivo organs (*n* = 3) at 24 h after 0.2 mg per mice i.v. injection. Organs: 1, tumor, 2, lung, 3, spleen, 4, brain, 5, liver, and 6, heart.***P < 0.001, **P < 0.01 compared with the organs of the control animals.

Prior to the in vivo efficacy study, we first conducted an in vivo safety evaluation of the Cor‐sugars in zebrafish embryos. As a well validated method for in vivo toxicity assessment, an AB (Danio rerio, AB strain) strain zebrafish were used and the larvae at 3 d after fertilization (3 dpf, days post fertilization) were collected for maximum tolerated dose (MTD) determination and morphological safety analysis.^[^ ^37^ ^]^ DOX was chosen as a positive control since DOX‐induced toxicity (cardiomyopathy, kidney injury etc.) is one of the most common toxicity diagnoses in zebrafish. The MTD was defined as a lethal concentration or doses that cause any adverse effect on the embryo development. DOX (0.1–20.0 ng per fish) and Cor‐sugars (2.2–40.0 ng per fish) were given via intravenous microinjection into each larva in the testing groups (2 nL per fish). Drug treated embryos were cultured in 6‐well plates at 30 embryos per well for 48 h. The PBS‐treated group and PBS containing 0.5% dimethyl sulfoxide (DMSO)‐treated embryos were used as two control groups. The organ‐specific toxicities were evaluated during the experiment, namely, abnormal heart rate, arrhythmia, abnormal circulation, pericardial edema and abnormal heart chamber morphology were used to evaluate the standard of cardiac toxicity; misshapen brain was analyzed for central nervous system toxicity; liver size and color were assessed for hepatotoxicity; and cyst formation and trunk edema were evaluated for renal toxicity. As summarized in **Figure** **5**a (Table S4 and Figure S33, Supporting Information), the MTD for DOX was recorded as 15 ng per fish (13.8 × 10^−6^ m , FW 543.52) due to the severe cardiac and renal edema observed at this concentration. Compared to DOX, the MTD for Cor‐sugars was found to be three‐fold higher in concentration at 40 ng per fish (41.7 × 10^−6^ m , FW 479.48). Despite the equally strong cytotoxicity of Cor‐sugars in cancer cell lines, the safety profiles observed from the MTD evaluation reflects the significant difference between DOX and the Cor‐sugars.

### 2.9 Cor‐Sugars Demonstrate Superior Efficacy in A549 Xenotransplant Zebrafish Model

Following the safety evaluation test, we conducted an antitumor efficacy study by using A549‐bearing zebrafish xenograft model. CM‐Dil fluorescent dye was used for A549 cell labeling and a total of 200 CM‐Dil‐labeled cancer cells in 15 nL PBS were microinjected into 2 pdf (days post fertilization) larvae and maintained in Holt buffer solution at 35 °C for 24 h (3 dpf) to finish the xenotransplant zebrafish model. For Cor‐sugars, two different doses at 10 and 20 ng per fish, and as positive control, 10 ng per fish for DOX were administered through, i.v., microinjection with PBS treated larvae as control group. After drug injection, the larvae were cultured at 35 °C for 48 h. At the end of the drug treatment, 10 larvae were randomly selected, anaesthetized with 0.016% (w/v) tricaine and imaged under a fluorescence microscope (AZ100, Nikon, Japan) equipped with a DP2‐BSW digital camera (Olympus, Inc.). Tumor cell fluorescence images were taken and FI was analyzed using NIS‐Elements D 3.10 software (The Nikon NIS‐Elements platform). Tumor inhibition rates were calculated according to the equation of tumor growth inhibitory (TGI) (%) = (FI of control group – FI of drug treated group)/FI of control group × 100%. The animal experiments were performed in accordance with the approval from the Chinese Academy of Medical Sciences, and all studies were performed in accordance with the approved guidelines from the China Zebrafish Resource Center. As shown in Figure 5b,c and Figure S34, Supporting Information, all Cor‐sugar compounds were able to inhibit the growth of A549 cells in a dose‐dependent manner. Among all tested compounds, Cor‐gal at 20 ng per fish treatment markedly inhibited the growth of A549 cells in a time‐ and dose‐dependent manner. After treatment for 48 h, the inhibitory rate of Cor‐gal was observed as 45%, and the highest efficacy for DOX at its MTD was 38%.

### 2.10 Cor‐Gal Inhibits Tumor Growth in A549 Xenograft Mice

On the basis of our in vivo data from zebrafish models, we further evaluated the antitumor effect of the most active compound Cor‐gal in A549 xenograft mouse model. As an evidence of selective tumor accumulation of Cor‐gal in rodent xenograft models, we first carried out a simple test using A549 bearing nude mice by giving a single dose of Cor‐gal at 0.2 mg mouse^−1^ by i.p. injection (equal to 10 mpk) with measurements of tissue distribution of the drug molecule after 24 h. As summarized in Figure 5d,e, the ex vivo imaging results showed that Cor‐gal abundantly distributed in tumor stroma but rarely accumulate in other tested normal organs except for the brain. The moderate accumulation of Cor‐gal in the brain, suggesting that Cor‐gal can penetrate the blood–brain barrier which is a well‐known GLUT1 overexpressing semipermeable border for brain nutrient uptake.^[^ ^38^ ^]^ This result provide additional evidence of Cor‐sugars for GLUT1 targeted tissue distribution.

**Figure 6:**
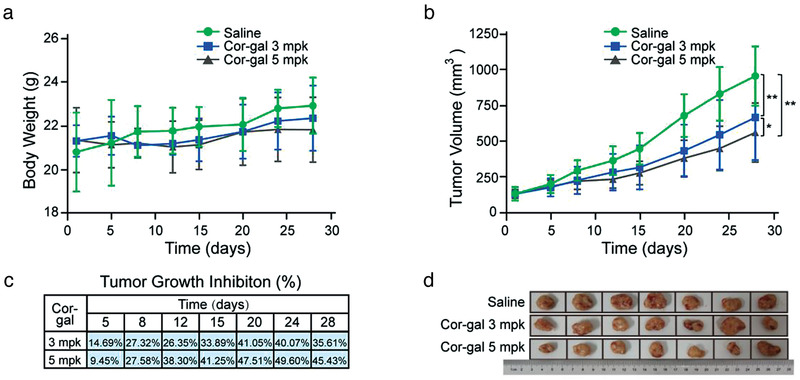
In vivo antitumor effects of Cor‐gal in A549 human lung cancer xenograft models. a) Body weight monitoring of the animals during efficacy studies. b) Tumor volume (mm^3^) of A549 after i.p. injection of PBS and Cor‐gal at the dosage of 3 and 5 mpk following 4 injection regimen on days 1/5/9/13. Data are presented as mean s.e.m. (standard error of mean) (*n* = 7 mice per group). **P < 0.01, *P < 0.05 compared with the control animals. c) The tumor growth inhibition of Cor‐gal treated mice based on the tumor weight. d) Images of excised tumors from each mouse in each treatment group.

As presented in **Figure** **6**a–d, with a four i.p. injection treatment regimen on days 1/5/9/13, Cor‐gal at 3 and 5 mpk administration, significantly suppressed the tumor growth of A549. The high TGI was recorded as 45–50% after 7–14 d observation period (Figure 6b,c). This result roughly aligns with the observations from the zebrafish efficacy studies although there is irresolvable issue on equivalent dose conversion between the two animal species.^[^ ^39^ ^]^ There was no body weight decrease in the mice during treatment indicating good tolerability of the animals and reflecting the intrinsic safety profile of Cor‐gal that we demonstrated in zebrafish and cell cycle alteration studies (Figure 6a) .

### 2.11 Cor‐Gal in Combination with Antiprogrammed Death‐Ligand 1 (PD‐L1) Antibody Eradicates H460 Human Lung Carcinoma

**Figure 7:**
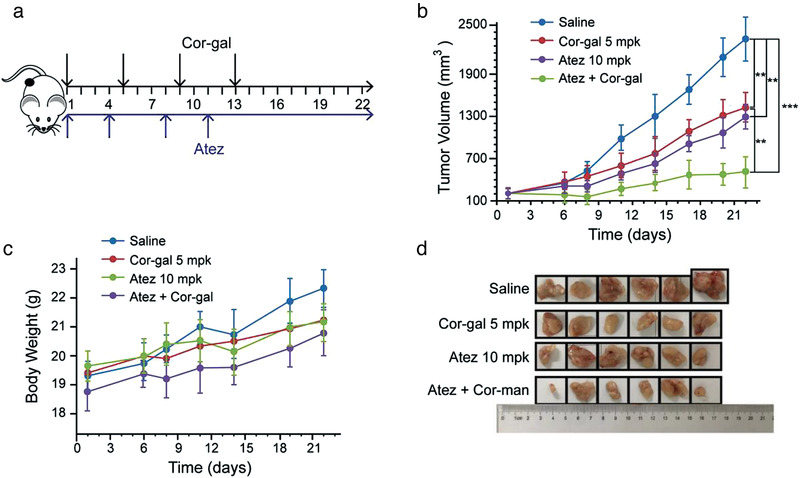
Cor‐gal in combination with CPI Atezolizumab eradicates H460 lung cancer in xenograft mice. a) The experimental schedule of the efficacy study. b) Tumor growth inhibition for each treatment groups. ***P < 0.001, **P < 0.01 compared with the control animals. c) Body weight monitoring of the animals during efficacy studies. d) Images of excised tumors from each mouse in each treatment group. Data are presented as mean s.e.m. (*n* = 6 mice per group).

Since Cor‐sugars are cell‐cycle dependent cytotoxic agents and exhibited in vitro and in vivo anticancer activities toward human lung cancer cells, based on the impedance efficacy of Cor‐gal in A549 xenograft tumors, we hypothesized that a dual‐targeting combination treatment of Cor‐gal with an immune CPI would show a greater therapeutic effect. To prove this hypothesis, instead of A549 cells, we selected H460 human lung cancer cells which also showed sensitivity against Cor‐sugars (Figure 2a), especially, H460 has been well studied to highly overexpress cytotoxic T lymphocyte antigen 4 (CTLA4) and PD‐L1, both as checkpoint proteins play key roles in promoting H460 proliferation and tumor growth.^[^ ^40^, ^41^ ^]^ To test the dual‐targeting hypothesis, we selected Atezolizumab as the anti‐PD‐L1 antibody as it has most widely used in the clinic. Atezolizumab is used in combination with cisplatin and etoposide for firstline treatment of patients with extensive‐stage small cell lung cancer, however, Atezolizumab mono therapy exhibits only weak to moderate tumor growth inhibitory effect in H460‐bearing mice (TGI: <40%)^[^ ^41^ ^]^ and do not show antitumor efficacy in A549 xenograft model (TGI: −4%).^[^ ^40^ ^]^ Based on these information, we examined the antitumor efficacy of Cor‐gal in combination with Atezolizumab using the H460 lung cancer model. The treatment schedule is shown in **Figure** **7**a. After tumor size reached to 100–150 mm^3^, 5 mpk of Cor‐gal was administered via i.p. injection on days 1/5/9/13, Atezolizumab antibody was administered via i.v. injection at a dosage of 10 mg kg^−1^ twice weekly for two weeks. The results were summarized in Figure 7b–d. The antitumor effect for Cor‐gal single agent treatment was similar with the above described in A549 model, the highest TGI was observed as 44.9%. For Atezolizumab, four injections achieved 49.2% for TGI in this study which is better than the reported results. The difference may due to the different animal model (H460‐bearing PBMCS‐CDX mice vs our nonobese diabetic/severe combined immunodeficiency (NOD‐SCID) mice) or simply comes from the different watching period (30 d vs 22 d). Importantly, synergies from the cell cycle arrest effect of Cor‐gal with PD‐L1 inhibitor exhibited an excellent antitumor effect that resulting in a near complete eradication of H460 (up to 90.8% for TGI).

### 2.12 Cor‐Gal Inhibits Cancer Cell Migration and Metastasis

**Figure 8:**
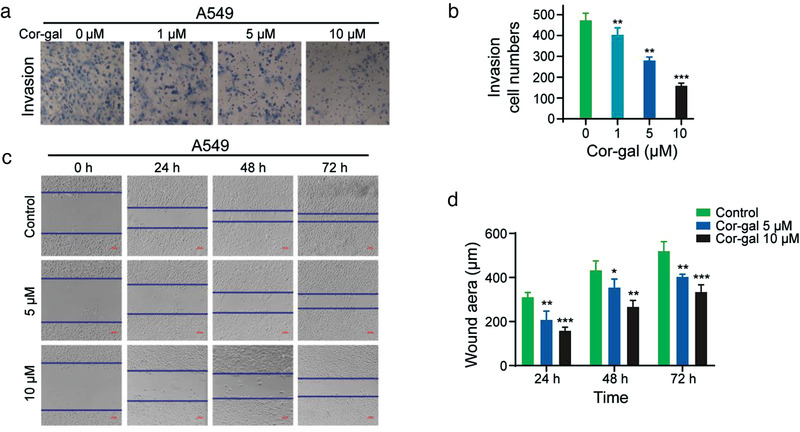
Cor‐gal exhibits the inhibitory effect on A549 cell migration and invasion. a) Representative images of A549 cells obtained after transwell cell invasion assay treated with different concentrations of Cor‐gal. b) Quantification of inhibition effect of the cell invasion assay. c) Wound healing assay of A549 cells upon Cor‐gal treatment (5 × 10^−6^ or 10 × 10^−6^ m) for different time period. The A549 cells treated with vehicle were used as control; d) Quantitation of the wound healing area in control and Cor‐gal treated cells. Significant difference with *p < 0.05, **p < 0.01, ***p < 0.001 versus Control.

To further probe the molecular aspects of Cor‐gal's anticancer mechanism, we tested if the Cor‐sugar compounds may have any inhibitory effect on tumor cell migration and invasion.^[^ ^42^ ^]^ First, A549 Matrigel invasion assay was performed using a trans‐well system. For membrane coating, 5 × 10^5^ A549 cells were seeded in the trans‐well chambers which were precoated with Matrigel (BD, USA) at 4 °C overnight and incubation with Matrigel solution at 37 °C for 30 min.^[^ ^42^ ^]^ After 72 h drug incubation with Cor‐gal, cells on the top surface were removed through using a cotton swab, and migrated cells on the basal side of the membranes were treated with paraformaldehyde at final concentration 4% in culture medium and with gentle washes with sterile 1X PBS to remove debris, nonattached cells, and fixation solution excess. As the result, Cor‐gal significantly suppressed A549 cancer cell invasion through the Matrigel membrane (**Figure** **8**a). Especially at the concentrations of 5 × 10^−6^ and 10 × 10^−6^ m , ≈40% and 66% suppression of cell invasion have been inhibited (Figure 8b). Follow the same procedure (without Matrigel coating), in the wound healing collective migration assay, migration of A549 cells was remarkably inhibited by treatment of Cor‐gal in a concentration‐ and time‐dependent manner (Figure 8c,d). As the most pronounced inhibitory effect, Cor‐gal decreased the ability of A549 cells on migration by ≈47% at 10 × 10^−6^ m for 24 h, compared with DMSO control (Figure 8d). The inhibitory effects observed with Cor‐gal on cancer cell invasion and migration implicate that more complex biological pathways may involve in the down‐ or upstream regulation of the anticancer process which is worthy for further investigations.

## 3 Discussion

Building large structures with curved corannulene surface or discover new functions with corannulene‐based self‐assembly are mainstream in corannulene research.^[^ ^2^, ^43^, ^44^ ^]^ However, challenges on deciphering cellular and molecular aspects as well as biological interactions of corannulene scaffold still remain. With our rationally designed Cor‐sugars, we have discovered some new insights on biological links between the single molecule of corannulene monosaccharides and tumor in cells. Based on the strategy of our molecular design, by installing triazolyl monosaccharide onto the corannulene core, we were able to make the molecule that with enhanced solubility to exert improved cellular uptake and thus Cor‐sugar became bioavailable. The conjugation of glucose, mannose and galactose endows the compound with biological functions such as recognition and absorption by cancer specific glucose transporters for selective targeting of hyperglycolytic tumors. The extended *π*‐conjugation system with triazole substitution enabled the Cor‐sugars to have enhanced fluorogenic performance for luminescence‐based in vitro and in vivo self‐probing, which greatly help the mechanistic studies in different biological events (Figure 1).

From confocal cell imaging study self‐probed by the compounds, we observed GLUT1 dependent cell uptake and cell nuclei distribution of Cor‐sugars (Figure 2). These results raise up the hypothesis that the potent cytotoxic activities of Cor‐sugars on a panel of human cancer cell lines (Figure 2) might attribute to their interactive binding capacity with the DNA which we have anticipated at the design stage, because the *π*‐electron rich corannulene have been demonstrated to be a suitable *π*‐*π* stacking moiety with other *π*‐electron structures, although there was no reported evidence on DNA interactions.^[^ ^45^, ^46^ ^]^ In fact, comparison studies with DNA targeting clinical drugs, we observed that Cor‐sugars could effectively suppress cancer cell proliferation with broad spectrum and superior activity than cisplatin (CDDP), carboplatin and DOX. Especially, Cor‐gal and Cor‐man exhibited very high sensitivity against the human lung cancer A549, H460, and the hormone‐insensitive human prostate cancer DU145 with comparable or superior cytotoxic activity to CDDP and DOX (Figure 2 and Tables S1–S2, Supporting Information). Before investigating the mechanistic details in DNA binding, we extend the survey to tumor selectivity and safety analyses. In cell level, Cor‐sugars show no detectable toxicity to the corresponding normal cell line BEAS‐2B (vs A549 cancer cell, Table S1, Supporting Information). In vivo, despite the equally strong cytotoxicity in cancer cell lines, Cor‐sugars displayed more safety profile than DOX in the maximum tolerated dose determination studies using zebrafish toxicology model (Figure 5). The selectivity of Cor‐sugars toward cytotoxic damage in GLUT1 overexpressing cancer cells can be reasonably explained from the GLUT1 dependent drug uptake results due to the Warburg effect mediated hyperglycolytic metabolism in tumor cells. In addition, our cell cycle arrest assay has revealed more exciting secrets for understanding the unexpected role of Cor‐gal in selective tumor annihilation. That is, in the normal BEAS‐2B cells, Cor‐gal treatment did not alter the distribution of the cells in cell cycles (Figure 4c–f). However, DOX treated normal cells still induced significant cell cycle alteration and cell cycle arrest. This results testifying the differences between Cor‐gal and DOX in cell cycle impairment effect and providing key insights into the unexpected role of Cor‐sugars in selective tumor targeting and their intrinsic safety profiles. Given the high in vitro anticancer activity and selectivity performance of Cor‐sugar compounds, we further testified their in vivo potency in three different xenograft models. First, in A549 bearing zebrafish larvae, Cor‐glu, Cor‐man, and Cor‐gal all exerted excellent anticancer efficacy at nontoxic doses, in which Cor‐gal exhibited superior tumor suppression results at 50% MTD dose over DOX at its MTD dose (Figure 5). Taking Cor‐gal as the representative compound, in A549 bearing xenograft mice model, four i.p injections at every 4 d of Cor‐gal at 3 and 5 mpk achieved 45–50% in TGI (after 7–14 d observation period) (Figure 6). As the third in vivo experiment, take the advantage of the cell cycle dependent action of Cor‐gal, a combination therapy of Cor‐gal with immune checkpoint inhibitor Atezolizumab, revealed a near complete eradication of H460 lung cancer in NOD‐SCID mice (Figure 7).

To decipher more details about the DNA binding mechanism of Cor‐sugars, we conducted a series of surveys on DNA interactions of the compounds, including the DNA‐mediated fluorescence quenching, binding affinity measurement, DNA hydrodynamic mobility, as well as MD simulation studies. First, the DNA‐binding parameters of Cor‐gal from ITC analysis revealed that the instant DNA binding of Cor‐gal is primarily driven by both the favorable binding entropy and enthalpy indicating that the binding process is basically dominated by the classical hydrophobic effect of the corannulene core. The ITC results also revealed that Cor‐gal binding can result in higher conformational disorder of DNA which may contribute to the stronger DNA unwinding potency as reflected by the increased entropy and enthalpy values (Figure 3c). With the DNA viscosity measurement, the results further clarified the interaction modes between DNA and the corannulene *π*‐bowls. Especially, after a long time scale incubation, Cor‐sugars induce large increases in relative viscosity of buffered DNA aqueous solution, although Cor‐glu exhibited a little weaker effect compared to Cor‐gal and Cor‐man. This observation provides proof that the curved corannulene *π*‐bowls can be typical intercalators as the intercalative binding is known to increase the hydrodynamic length and leading to an increase in the DNA viscosity. In accordance with the ITC results, instant incubation of increasing amount of Cor‐sugars produces negative viscosity changes which is indicative of Cor‐sugars in groove binding because the DNA bending induced by groove binders will results in a decrease of its effective length and, concomitantly, the viscosity. Interestingly, this binding mode can be detected up to 24 h in the Cor‐sugar/DNA mixture. Generally, as a DNA‐targeting agent, higher DNA binding affinity and slow DNA binding kinetics are considered ideal characteristics although this remains a big challenge in practice. Since stronger DNA‐binding may maximize the efficacy with minimal drug exposure, and lower association rates enable drug molecules to target specific sequences, and ensure disruption of both DNA transcription and duplication.^[^ ^47^ ^]^ Our preliminary survey has confirmed the fact that Cor‐gal possesses weaker instant binding affinity but exhibits stronger DNA damage responses. This phenomenon can be explained by the time dependent and multiphasic DNA binding property of the compound which deduced from the viscosity studies.

## 4 Conclusion

In summary, to discover the biomedical property of fulleretic corannulene molecule, sugar‐conjugated triazolylcorannulene *π*‐bowls were rationally designed and synthesized. The anticancer activity of the Cor‐sugars and underlying biological mechanisms including the targeted cell uptake, cell cycle arrest and tumor cell metastasis inhibition were comprehensively evaluated. Among the Cor‐sugars investigated, Cor‐gal exhibits the most effective anticancer potential in A549 zebrafish and athymic mice xenograft models. The synergistic antitumor effect of Cor‐gal combined with immune checkpoint inhibitor Atezolizumab for dual targeted therapy in H460 treatment was found to be a very effective strategy to defeat the highly aggressive and metastatic lung cancer. Self‐probed cellular uptake studies revealed that Cor‐sugars are able to be transported through GLUT1 for targeted tumor cell delivery and intratumoral accumulation by leveraging the cancer specific Warburg effect. The extraordinary safety profile of Cor‐sugars relies on the unique property of the molecule to selectively induce cell cycle arrest in tumor cells without harming the normal cells. The enhanced anticancer efficacy was attributed at least to the molecular function of Cor‐sugars in DNA damage response with a unique multiphasic DNA binding mechanism. To the best of our knowledge, this is the first example to uncover the unexpected role of corannulene buckybowl in selective tumor targeting and explore their potential new applications in biomedical engineering. Although other underlying biological mechanisms need to be further investigated in more detail, through the current study, we identified that the bowl‐shaped corannulene derivatives may also be an important class of DNA‐interacting antitumor agents. Thus, the corannulene *π*‐bowl‐based analogs could represent a new approach to DNA‐transcription targeted drug design with very useful biological and pharmacological activities.

## 5 Experimental Section

### Chemicals and Operations

All chemical reagents for synthesis of Cor‐sugars were obtained from commercial suppliers and used as received. pBR322 plasmid DNA and Herring sperm DNA (hsDNA) was purchased from Solarbio and stored at 2–8 °C. Atezolizumab was provided by Tianjin Medical University Cancer Institute. Cor‐glu, Cor‐gal, and Cor‐gal were dissolved in 100% DMSO to make a 10–50 mg mL^−1^ parent solution. DOX was dissolved in 100% DMSO to make a 5 mg mL^−1^ solution. To obtain the desired concentration, before each experiment, the Cor‐sugars were diluted with PBS (for cell assays), Tris‐HCl buffer (pH 7.2, for DNA interaction analysis), or E3 embryo media (for zebrafish studies) at 45 °C under ultrasonication for 15 min for complete drug dissolution. The final DMSO concentration never exceeded 0.5% v/v. All drug solutions for biological assay were prepared just before use.

### Cell Culture

All cells were purchased from american type culture collection (ATCC), China. Human colon cancer (HT29), human prostate cancer (DU145), human cervical cancer (HeLa), HGC27, human multiple myeloma (H929), and human lung cancer (H460 and A549) cell lines were cultured as an adherent monolayer in roswell park memorial institute culture medium (RPMI)‐1640, HEK 293, and human hepatocellular carcinoma (HepG2) cells were cultured in Dulbecco's modified Eagle's medium (DMEM, Gibco) supplemented with 1% w/v glutamine and 10% v/v heat‐inactivated fetal bovine serum (FBS, Gibco) at 37 °C under a humidified atmosphere containing 5% CO_2_ and 95% air.

### Cytotoxicity Assay

Cells were cultured to reach ≈80–85% confluence prior to drug treatment. Cells were seeded in a 96‐well flat‐bottomed microplate in 100 µL of the corresponding growth medium and were treated with either vehicle or the test compounds (DOX, carboplatin, Cor‐glu, Cor‐gal and Cor‐gal). The microplate was incubated at 37 °C under 5% CO_2_ and 95% air in a humidified incubator for 72 h. MTT (Sigma‐Aldrich) was added to each well at a final concentration of 0.83 mg mL^−1^ and incubated for 2 h. The cells were lysed by sodium dodecyl sulfate lysis buffer (2% SDS, 50 × 10^−3^ m Tris‐HCl) and the absorption of MTT formazan was measured at 570 nm using a multi‐well‐reading UV‐Vis spectrometer. For each compound, the cell survival rate was expressed as the relative percentage of absorbance compared to that of the control sample without drug. The IC_50_ values of each test compound as concentration required to reduce the absorbance by 50% compared to the controls, were calculated according to the optical density (OD) value. Each experiment was assessed three times with five replicates (five wells of the 96‐well plate per experimental condition).

### Cellular Uptake and Intracellular Accumulation

Confocal laser scanning microscopy was applied to investigate the cellular uptake and intracellular accumulation of the Cor‐sugars. A549 cells (1×10^5^ cells) were grown on a Lab‐Tek II chambered coverglass (Thermo Scientific Nunc) and allowed to adhere for 24 h under normoxic conditions. After incubation with 50 × 10^−9^ m Cor‐sugars at 37 °C for 30 min, the drug‐containing solutions were removed, and 1.0 mg mL^−1^ propidium iodide (PI, Solarbio) was added and incubated for another 5 min. After the staining solution was removed, the cells were rinsed three times with PBS, and DNase‐free RNase (freshly made) was added to a final concentration of 100 µg mL^−1^ and incubated at 37 °C for 30 min. Then, cell imaging was conducted on an Olympus FV1000‐IX81 confocal laser scanning microscope through a 100 × 1.4 numerical aperture oil immersion objective lens (Olympus). All parameters of the microscope were set to be the same for different samples to allow comparisons among the Cor‐sugars.

### GLUT1 Transfected Cell Assay

*HEK‐293‐GLUT1 Mediated Drug Uptake Assay*: Stable transfection and the preparation of GLUT1 overexpressed cell line HEK‐293FT‐GLUT1 were followed the previous published procedures (21). 293FT‐GLUT1 and 293FT cells were seeded in 96‐well black and clear‐bottomed culture plates. After overnight incubation, culture media was changed to glucose‐free DMEM (100 µL per well) and incubated at 37 °C for 2 h. After remove of growth medium, cells were treated with 100 µL of different concentration Cor‐sugar solution (0.1 × 10^−6^, 1 × 10^−6^, 2 × 10^−6^, 5 × 10^−6^, 10 × 10^−6^ , 20 × 10^−6^ , and 25 × 10^−6^ m ) for 40 min. Cells were washed with cold PBS three times and subjected to fluorescence detection (λex = 295 nm). Background measurements were taken using glucose‐free DMEM. For quantitative purposes, cells were subjected to the MTT‐mediated cell viability assay to normalize the fluorescence levels according to the number of living cells.

### GLUT Inhibitor‐Dependent Cellular Uptake

A549 cells were seeded in 96‐well black and clear‐bottomed culture plates. After overnight incubation, culture media was changed to glucose‐free RPMI 1640 (100 µL per well) and incubated at 37 °C for 2 h. After remove of growth medium, cells were treated with 100 µL of the GLUT inhibitor Phloretin at final concentration of 0.1 × 10^−3^ m and incubated for 30 min. Then different concentration of Cor‐sugar solution (0.1 × 10^−6^, 1 × 10^−6^, 2 × 10^−6^, 5 × 10^−6^, 10 × 10^−6^, 20 × 10^−6^, and 25 × 10^−6^ m) was added and incubated for 40 min. Cells were washed with cold PBS for three times and subjected to fluorescence detection (lex = 295 nm). Background measurements were taken using glucose‐free RPMI 1640. For quantitative purposes, cells were subjected to the MTT‐mediated cell viability assay to normalize the fluorescence levels according to the number of living cells.

### CD Spectroscopy

CD measurements were performed on a Jasco J‐810 spectrophotometer. CD spectra of 5 × 10^−6^ m hsDNA (Solarbio, D8050) in the absence and presence of varying concentrations of compounds (0–15 × 10^−6^ m) were recorded with a rectangular quartz cell having a 5.00 mm pathlength. The CD measurements were performed in Tris‐HCl buffer (Solarbio, T1080, 150 × 10^−3^ m NaCl, pH 7.2) under constant nitrogen flush at room temperature after 12 h incubation. Samples for interaction studies over different times were prepared by incubating samples at 25 °C. All observed CD spectra were corrected by subtracting the buffer signal.

### Isothermal Titration Calorimetry

hsDNA was dialyzed overnight against 150 × 10^−3^ m KCl, 10 × 10^−3^ m N‐2‐hydroxyethylpiperazine‐N'‐2‐ethanesulfonic acid (HEPES) (pH 7.4 containing 0.5% DMSO) at 4 °C. The compound Cor‐gal was dissolved to 5 × 10^−6^ and 10 × 10^−6^ m in the same buffer used for DNA. Solutions were degassed by ultrasonic device and centrifuged at 15 000 × *g* for 10 min before titration. Then Cor‐gal solution was added into the sample cell and the DNA solution was loaded in the syringe for titration. The ITC experiment was performed with a MicroCal PEAQ‐ITC instrument (Malvern Instruments, Great Britain) at 25 °C at the time intervals of 150 s and a stirring speed of 750 rpm. 100 × 10^−6^ m hsDNA was conducted by 13 aliquots and titrated into the calorimetry cell containing either Cor‐gal or control buffer. The first injection of 0.4 µL DNA was ignored to eliminate the diffusion effects from the syringe on the sample cell, and the following injections were carried out at 3 µL. The cell filled with dialyzed buffer titrated by DNA was assigned as control to get the blank data. Final data were analyzed with the MicroCal PEAQ‐ITC analysis software. The “one set of binding sites” fitting model was applied to analyze the thermodynamic parameters.

### DNA Viscosity Measurement

Viscosity measurements were carried out using a calibrated Ubbelohde viscometer (F 0.5–0.6 mm, Shanghai Qianfeng Rubber and Glass Company, Shanghai, China) immersed in a thermostatic water bath maintained at 25 ± 0.1 °C. The hsDNA concentration was kept constant (5 × 10^−6^ m), while the complex concentration was varied at [Cor‐sugar]/[DNA] (*r*) ratios in the range of 0.0–0.4. The viscometer was cleaned by washing with distilled water and sodium chloride solution and drying in acetone. Samples were pipetted into the reservoir of the viscometer immersed in a water bath set at 25 ± 0.1 °C. The efflux time was then measured with a digital stopwatch. Each sample was measured three times and the average flow time was recorded. The relative viscosities of hsDNA in the presence and absence of the complex were calculated from the relation *ƞ* = (*t* − *t*_0_)/*t*_0_, where *t* is the observed flow time of DNA‐containing solution and *t*_0_ is the flow time of Tris buffer alone. Data are presented as (*ƞ*/*ƞ*_0_)^1/3^ versus binding ratio, where *ƞ* is the viscosity of hsDNA in the presence of compounds and *ƞ*_0_ is the viscosity of hsDNA alone.

### Cell Cycle Assays

A549 and BEAS‐2B cells were seeded in six‐well plates at a density of 1 × 10^5^ cells mL^−1^ and cultured for 24 h. Cor‐gal and DOX were added to cells at the dosage of 3 × 10^−6^ m incubated for another 24 h. Then the cells were harvested, washed with cold PBS, and resuspended in cold ethanol to a final concentration of 70%. Subsequently, the cells were fixed at −20 °C overnight. For cell cycle analysis, the fixed cells were washed with PBS and treated for 1 h at 37 °C with RNase (100 µg mL^−1^) prior to PI staining (10 µg mL^−1^ in the dark for 10 min) and immediately analyzed by flow cytometry.

### Western Blot Analysis

Western blot analysis was performed as described previously (20) using the following antibodies: GLUT1 (cat. no. ab652, Abcam), Phospho‐Histone H2A.X (Ser139) (cat. no. 9718T, Cell Signaling Technology), and anti‐*β*‐actin antibody (cat. no. 4970S, Cell Signaling Technology).

### Cell Invasion Assay

Cell invasion of A549 cells were measured using transwell cambers with 8 µm pores and transwell membranes precoated with Matrigel (BD Bioscience). Cells (1 × 10^5^ cells per well) were seeded in upper chambers of transwell plates in serum‐free‐DMEM medium. To the lower chambers of the plate, 0.6 mL DMEM containing 10% FBS was added as attractant. Cor‐gal at the dose of 5 × 10^−6^, 10 × 10^−6^ m, and DMSO control were added into the upper chamber. After 48 h incubation, invasive cells in the lower chambers were fixed with 4% formaldehyde for 15 min and stained with 2% crystal violet for 15 min. After being washed by PBS for three times, the invasion rate was measured by counting the migrating cells under the microscope.

### Wound Healing Assay

A549 cells were seeded into a six‐well plate at concentration 5 × 10^5^ cells per well. When the cells were cultured in DMEM media with 10% FBS until 90% confluent, the media were then replaced by PBS, and an artificially single scratch was created across the monolayer with a plastic 100 µL pipette tip held perpendicular to the plate bottom. After several PBS washes to remove floating cells and the cells were continuously cultured in the medium without FBS and different dose of Cor‐gal or control reagent. The wound areas were recorded at 0, 24, 48, and 72 h using a digital camera system. The migration distance was quantificated by the ImageJ software. Experiments were performed in triplicate and repeated three times.

### In Vivo Efficacy in A549 Xenograft Mice

A549 human lung cancer cells (1 × 10^6^) were prepared in vitro and subcutaneously injected into the right flank of 6–8 week old NOD‐SCID mice. Tumor volumes were monitored by caliper measurements following the formula of (length x width^2^)/2. Animals were divided into three groups when the tumor size reached 100–150 mm^3^: PBS control group, Cor‐gal (3 mpk) group and Cor‐gal (5 mpk) group. Each group contains seven animals. Drugs were administered via i.p. injection on days 1/5/9/13. Tumor sizes and body weight were monitored every three days. The animals were observed for another two weeks after the final drug injection. For in vivo studies, all animal experiments were performed according to the regulation and guideline of Institutional Animal Care and Use Committee at Tianjin Medical University Cancer Institute and Hospital by following the Guide for the Care and Use of Laboratory Animals (NRC 2011).

### In Vivo Combination Therapy in H460 Xenograft Mice

H460 human lung cancer cells (1×10^6^) were prepared in vitro and subcutaneously injected into the right flank of 6–8 week old female NOD‐SCID mice. Tumor volumes were monitored by caliper measurements following the formula of (length x width^2^)/2 and drug treatment was started when tumor reached 100–150 mm^3^ in volume. Mice were grouped as PBS control, Cor‐gal (5 mpk), Atez (stezolizumab, 10 mpk), Cor‐gal + Atez (*n* = 6). Cor‐gal was administered via i.p. injection as the same regimen used in A549 models, and Atezolizumab antibody was administered via intravenous injection at a dosage of 10 mg kg^−1^ twice weekly for two weeks. Animals were monitored after the final drug injection for days.

### Statistical Analysis

Statistical analysis for in vitro assay data was performed with GraphPad Prism 7 software and data were expressed as mean ± standard deviation. Significance of in vivo tumor growth comparisons was carried out using two‐sided *t*‐tests. Statistically significant differences between experimental groups were determined using GraphPad Prism 7 and as previously described in the studies.^[^ ^20^, ^21^, ^22^ ^]^ The symbols *, **, and *** throughout all figures indicate *P*‐values less than 0.05, 0.01, and 0.001, respectively.

## Conflict of Interest

The authors declare no conflict of interest.

## Supporting information

Supporting Information

## Data Availability

The data that support the findings of this study are available in the supplementary material of this article.
