# Supplementary material for: An Unrevealed Molecular Function of Corannulene Buckybowl Glycoconjugates in Selective Tumor Annihilation by Targeting the Cancer‐Specific Warburg Effect
Source: Adv Sci (Weinh). 2022 Mar 7;9(10):2105315. doi: 10.1002/advs.202105315 (PMC8981914; doi:10.1002/advs.202105315)
Supplement: Supplementary file 1 — Supporting Information [file ADVS-9-2105315-s001.pdf]

ADVANCED SCIENCE
Open Access

Supporting Information

for *Adv. Sci.*, DOI 10.1002/advs.202105315

An Unrevealed Molecular Function of Corannulene Buckybowl Glycoconjugates in Selective Tumor Annihilation by Targeting the Cancer-Specific Warburg Effect

Shengnan Liu, Ziru Sun, Min Liang, Weijie Song, Ru Zhang, Yunli Shi, Yujun Cui and Qingzhi Gao**

WILEY-VCH

Supporting Information

An unrevealed molecular function of corannulene buckybowl glycoconjugates in selective tumor annihilation by targeting the cancer-specific Warburg effect

Shengnan Liu †, Ziru Sun †, Min Liang, Weijie Song, Ru Zhang, Yunli Shi, Yujun Cui, Qingzhi Gao**

Table of Contents

1. Chemistry and materials
2. Fluorescence property
3. *In vitro* anticancer activity
4. GLUT-dependent cell uptake
5. Subcellular distribution of Cor-gal
6. DNA interaction/circular dichroism
7. DNA-binding mechanism/ viscosity analyses
8. Stability assessment of Cor-sugars
9. MD simulation study
10. *In vivo* toxicity in zebrafish
11. *In vivo* efficacy study in zebrafish xenograft

Figs. S1 to S15: All spectra information of Cor-glu, Cor-gal and Cor-man
Figs. S16 to S1:9 Fluorescence property data of corannulene, Cor-gal and Cor-man
Figure S20: GLUT-dependent cell uptake of Cor-man and Corgal
Figure S21: Subcellular distribution of Cor-gal in A549 cells
Figure S22 to S23: CD spectra of hsDNA titrated with Cor-glu and Corman
Figure S24: Relative viscosity changes of hsDNA treated with Cor-glu and Cor-gal
Figure S25 to S28: Stability analysis of Cor-glu, Cor-man and Cor-gal in assay buffer
Figure S29 to S32: 200 ns MD simulation results of Cor-gal with 15-mer DNA
Figure S33: Toxic phenotype data of DOX and Cor-sugars in zebrafish
Figure S34: In vivo efficacy results of Cor-sugars in A549 bearing zebrafish model

Table S1: Cytotoxicity of the Cor-sugars against different cancer cell lines
Table S2: Cytotoxicity of the Cor-sugars against A549 cancer cells
Table S3: Binding energies of Cor-gal with 15-mer DNA after 200 ns MD simulation
Table S4: MTD data of DOX and Cor-sugars in zebrafish

1. Chemistry and Materials

General. Part of the corannulene (COR) starting material was kindly provided by Prof. Jianhui Huang from Tianjin University. All other chemicals were obtained from commercial suppliers and were used as received. Herring sperm DNA (hsDNA) was purchased from Solarbio (D8050, Sigma) and stored at 2-8 °C. Details for the syntheses and characterizations of Cor-sugars are described in supplementary information. If necessary, the reactions were carried out in dry solvents and under an argon atmosphere. ^{1}H and ^{13}CNMR spectra were recorded with a Bruker Avance 400 or 600 MHz at the School of Pharmaceutical Science and Technology of Tianjin University, PRC. Data are reported as chemical shifts (δ) in parts per million (ppm) relative to the solvent peak, and scalar coupling constants (*J*) are reported in units of hertz (Hz). HPLC analyses were carried out using a CXTH-LC3000 analytical and semi-preparative gradient HPLC system with a DaisoGel C18 (4.6 x 250 mm, 5 mm) and DaisoGel C18 (20 x 250 mm, 10 mm) column at room temperature. The mobile phase consisted of MeOH and H_2O, and the flow rate was 1 mL/min for analytical purification and 15 mL/min for preparative purification. Infrared spectra were recorded using a Bruker Tensor 27 FT-IR spectrometer in a KBr pellet, which was prepared by grinding the solid sample with solid KBr and applying great pressure to the dry mixture. Absorptions are reported in wavenumbers (cm^{-1}). UV-visible spectroscopic analysis was performed using a U-3900 UV-VIS spectrophotometer at room temperature with a quartz cuvette having a pathlength of 0.2 cm as a sample holder. Fluorescence spectra were measured using a Varioskan LUX multimode microplate reader (Thermo Fisher Scientific). Viscosity measurements were carried out using an Ubbelohde viscometer immersed in a thermostatic water bath maintained at 25 ± 0.1 °C. Circular dichroism (CD) measurements were performed on a Jasco J-810 spectrophotometer. High-resolution mass spectra (HRMS, m/z) were recorded on a Bruker MicroTOF spectrometer in positive mode (ESI+). Real-time fluorescence quantitative PCR was performed on a QuantStudio™ 6 Flex Real-Time PCR system (Applied Biosystems).

Synthesis and characterization of sugar-conjugated corannulene *π*-bowls

The corannulene-sugar conjugates were synthesized according to the references of the total synthesis described in Fig. 2. Briefly, preparations for corannulene (1), corannulene bromide (2), and ethynylcorannulene (3) were followed the reported methods. The copper-catalysed “click” cyclization of ethynylcorannulene with sugar azides under aqueous conditions (t-BuOH/H_2O) allows the selective production of the desired triazolylcorannulene sugar-conjugates of glucose (Cor-glu), mannose (Cor-man) and galactose (Cor-gal) in high yield after deprotection of the acetyl groups on the pendant sugars. A detailed description of the synthetic procedures by which the Cor-sugars were synthesized, as well as their ^{1}H and ^{13}C NMR spectroscopy, UV, FT-IR, high-resolution mass spectrometry (HRMS) and HPLC purity data, is included in the Supplementary Information.

Preparation of glucose-conjugated triazolylcorannulene (b): Cor-glu

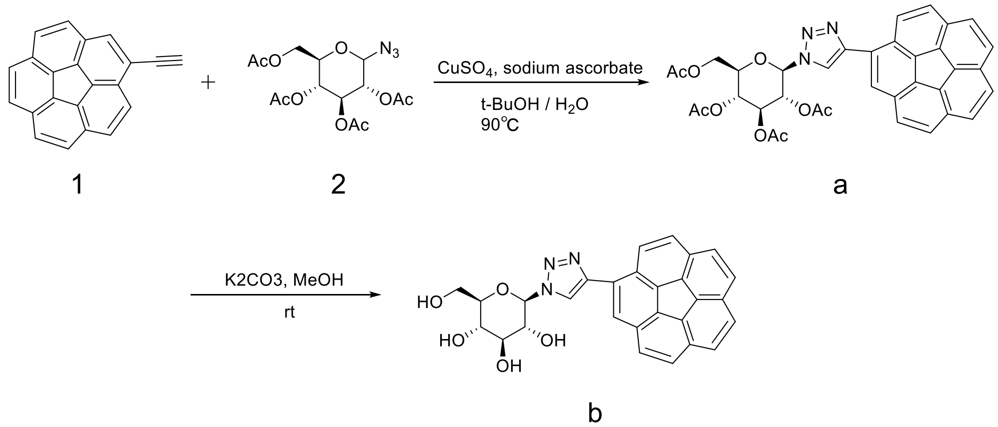

1-(2,3,4,6-tetra-O-acetyl-β-D-glucopyranosyl)-4-corannulenyl-[1,2,3]-triazole (a). To a solution of **1** (50 mg, 0.182 mmol) and **2** (75 mg, 0.201 mmol) in 4 mL of *t*-BuOH/H_2O (v/v: 1:1), $CuSO_4 \cdot 5H_2O$ (23 mg, 0.092 mmol), sodium ascorbate (73 mg, 0.377 mmol) were added and the reaction mixture was heated at 80 °C for 24 h. After cooling to the room temperature, 10 mL of water was added and the reaction mixture was extracted twice with 20 mL of CH_2Cl_2. The combined organic phases were dried over Na_2SO_4 and the solvent was removed under reduced pressure. The crude reaction mixture was purified by column chromatography using silica gel (PE: EA = 3:1) to give compound **a** as a colorless oil (95 mg, 85% yield).

Cor-glu: 1-(β-D-glucopyranosyl)-4-corannulenyl-[1,2,3]-triazole (b). a (95 mg, 0.167 mmol) was dissolved in a mixture of MeOH (4 mL). To this solution anhydrous K_2CO_3 (164 mg, 1.188 mmol) was added. The mixture was stirred at room temperature for 5 h and filtered. The crude reaction mixture was purified by column chromatography using silica gel (CH_2Cl_2 : MeOH = 10:1) to give compound **b** as a white solid(67.8 mg, 96.4%).

^{1}H NMR (400 MHz, DMSO) δ 9.16 (s, 1H), 8.55 – 8.48 (m, 2H), 8.08 – 7.98 (m, 7H), 5.70 (d, *J* = 9.2 Hz, 1H), 5.56 (d, *J* = 5.5 Hz, 1H, exchanges with D_2O, OH), 5.39 (d, *J* = 3.7 Hz, 1H, exchanges with D_2O, OH), 5.23 (d, *J* = 4.9 Hz, 1H, exchanges with D_2O, OH), 4.69 (t, *J* = 5.2 Hz, 1H, exchanges with D_2O, OH), 4.02 – 3.92 (m, 1H), 3.81 – 3.71 (m, 1H), 3.59 – 3.44 (m, 4H).

^{13}C NMR (100MHz, DMSO): δ 145.61 135.20, 135.08, 134.73, 134.60, 134.38, 130.81, 130.76, 131.56, 130.32, 129.39, 128.04, 127.98,127.89, 127.78, 127.54, 127.43, 127.31, 124.94, 122.73, 87.86, 80.55, 76.88, 72.14, 69.59, 60.78.

IR (KBr): 3693, 3300, 1697, 1398, 1072, 874, 826, 662 cm^{-1}.

HRMS: Calcd. For $C_{28}H_{21}N_3O_5$ $(M+Na)^+$: 502.1373, found: 502.1370.

Preparation of mannose-conjugated triazolylcorannulene (f): Cor-man

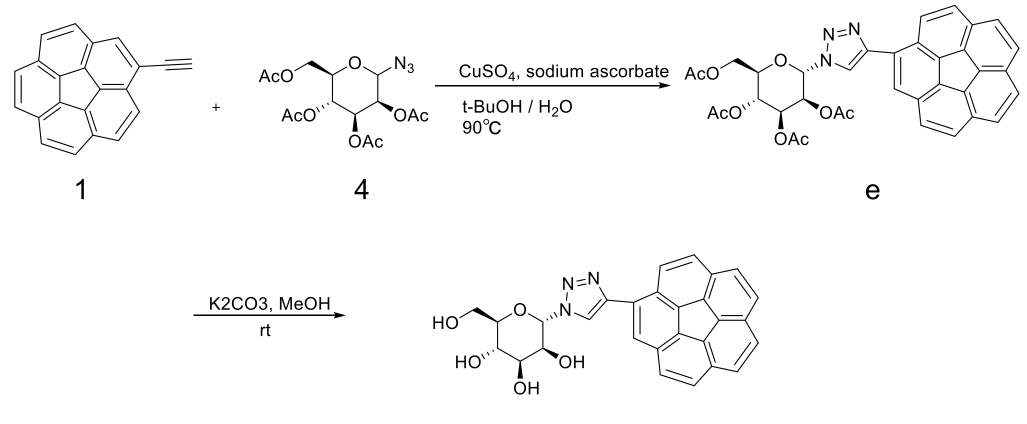

1-(2,3,4,6-tetra-O-acetyl-α-D-mannopyranosyl)-4-corannulenyl-[1,2,3]-triazole (e). To a solution of **1** (50 mg, 0.182 mmol) and **4** (75 mg, 0.201 mmol) in 4 mL of *t*-BuOH/H_2O (v/v: 1:1), $CuSO_4 \cdot 5H_2O$ (23 mg, 0.092 mmol), sodium ascorbate (73 mg, 0.377 mmol) were added and the reaction mixture was heated at 80 °C for 24 h. After cooling to the room temperature, 10 mL of water was added and the reaction mixture was extracted twice with 20 mL of CH_2Cl_2. The combined organic phases were dried over Na_2SO_4 and the solvent was removed under reduced pressure. The crude reaction mixture was purified by column chromatography using silica gel (PE: EA = 3:1) to give compound **e** as a colorless oil (100 mg, 89.5% yield).

Cor-man: 1-(α-D-mannopyranosyl)-4-corannulenyl-[1,2,3]-triazole (f). e (100 mg, 0.176 mmol) was dissolved in a mixture of MeOH (4 mL). To this solution anhydrous K_2CO_3 (172.6 mg, 1.224 mmol) was added. The mixture was stirred at room temperature for 5 h and filtered. The crude reaction mixture was purified by column chromatography using silica gel (CH_2Cl_2 : MeOH = 10:1) to give compound **f** as a white solid(68.6 mg, 97.6%).

^{1}H NMR (400 MHz, DMSO) δ 9.03 (s, 1H), 8.53 – 8.47 (m, 2H), 8.08 – 7.98 (m, 7H), 6.08 (d, J = 4.5 Hz, 1H), 4.59 – 4.56 (m, 1H), 3.96 – 3.92 (m, 1H), 3.72 – 3.62 (m, 4H).

^{13}C NMR (100MHz, DMSO): δ 146.05, 135.70, 135.59, 135.24, 135.09, 134.90, 131.32, 131.27, 131.06, 130.80, 129.83, 128.60, 128.56, 128.39, 128.30, 128.06, 127.85, 127.82, 125.51, 123.88, 86.51, 79.15, 72.96, 71.85, 68.58, 61.25.

IR (KBr): 3682, 3162, 1730, 1398, 1071, 826 cm^{-1}.

HRMS: Calcd. For $C_{28}H_{21}N_3O_5$ $(M+Na)^+$: 502.1373, found: 502.1370.

Preparation of galactose-conjugated triazolylcorannulene (d): Cor-gal

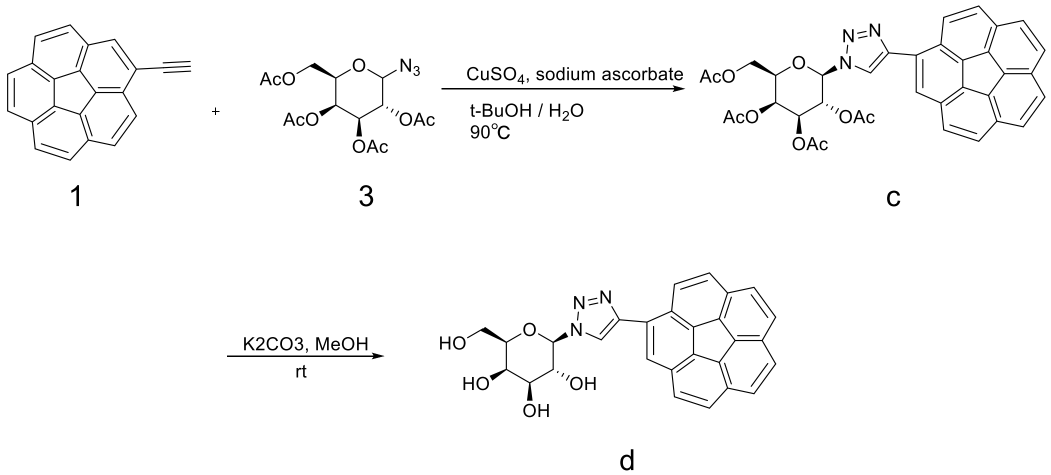

1-(2,3,4,6-tetra-O-acetyl-β-D-galactopyranosyl)-4-corannulenyl-[1,2,3]-triazole I. To a solution of **1** (50 mg, 0.182 mmol) and **3** (75 mg, 0.201 mmol) in 4 mL of *t*-BuOH/H_2O (v/v: 1:1), $CuSO_4·5H_2O$ (23 mg, 0.092 mmol), sodium ascorbate (73 mg, 0.377 mmol) were added and the reaction mixture was heated at 80 °C for 24 h. After cooling to the room temperature, 10 mL of water was added and the reaction mixture was extracted twice with 20 mL of CH_2Cl_2. The combined organic phases were dried over Na_2SO_4 and the solvent was removed under reduced pressure. The crude reaction mixture was purified by column chromatography using silica gel (PE: EA = 3:1) to give compound **c** as a colorless oil (93 mg, 83.21% yield).

Cor-gal: 1-(β-D-galactopyranosyl)-4-corannulenyl-[1,2,3]-triazole (d). c (93 mg, 0.163 mmol) was dissolved in a mixture of MeOH (4 mL). To this solution anhydrous K_2CO_3 (160.5 mg, 1.163 mmol) was added. The mixture was stirred at room temperature for 5 h and filtered. The crude reaction mixture was purified by column chromatography using silica gel (CH_2Cl_2 : MeOH = 10:1) to give compound **d** as a white solid(65.8mg, 93.5%).

^{1}H NMR (400 MHz, DMSO) δ 9.10 (s, 1H), 8.56 – 8.49 (m, 2H), 8.08 – 7.98 (m, 7H), 5.65 (d, *J* = 9.2 Hz, 1H), 5.41 (d, *J* = 5.9 Hz, 1H, exchanges with D_2O, OH), 5.11 (d, *J* = 5.6 Hz, 1H, exchanges with D_2O, OH), 4.75 (t, *J* = 5.7 Hz, 1H, exchanges with D_2O, OH), 4.69 (d, *J* = 5.6 Hz, 1H, exchanges with D_2O, OH), 4.28 – 4.20 (m, 1H), 3.85 – 3.79 (m, 2H), 3.68 – 3.53 (m, 3H).

^{13}C NMR (100 MHz, DMSO) δ 146.21, 135.71, 135.59, 135.25, 135.10, 134.88, 131.31, 131.26, 131.05, 130.86, 129.90, 128.57, 128.50, 128.37, 128.04, 127.92, 127.84, 127.81, 125.51, 122.97, 88.96, 79.10, 74.13, 69.91, 69.07, 61.01.

IR (KBr): 3679, 3144, 1696, 1384, 1089, 834, 705 cm^{-1}.

HRMS: Calcd. For $C_{28}H_{21}N_3O_5$ $(M+Na)^+$: 502.1373, found: 502.1370.

Characterization and Analytical Data of the Compounds

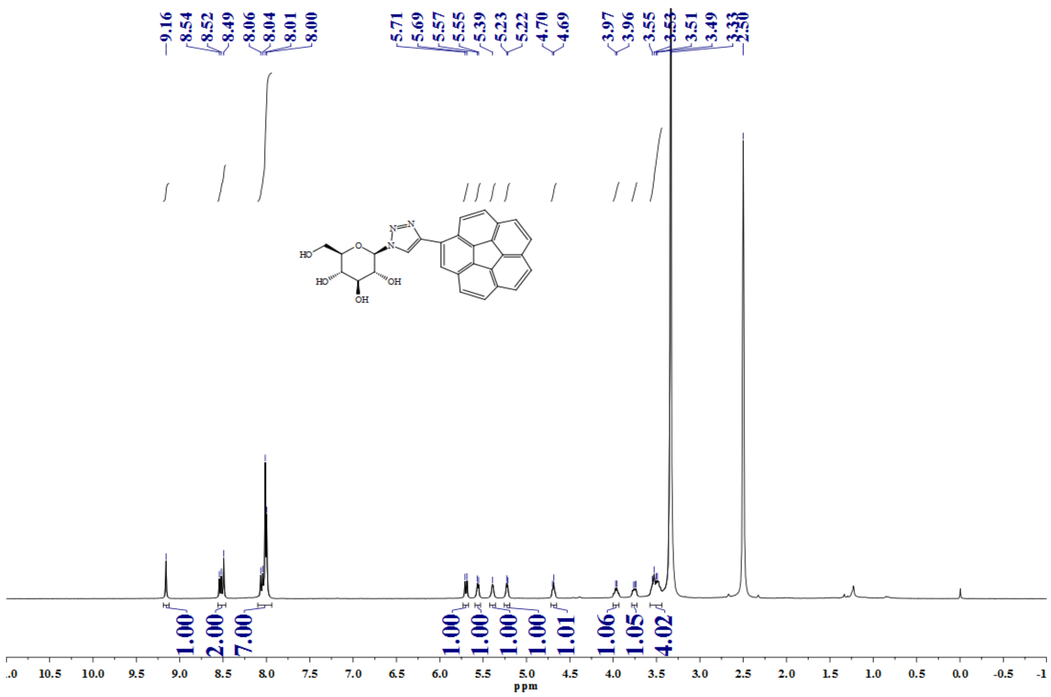

Fig. S1. ^{1}H NMR (400 MHz) spectrum of **Cor-glu** in DMSO-*d6*.

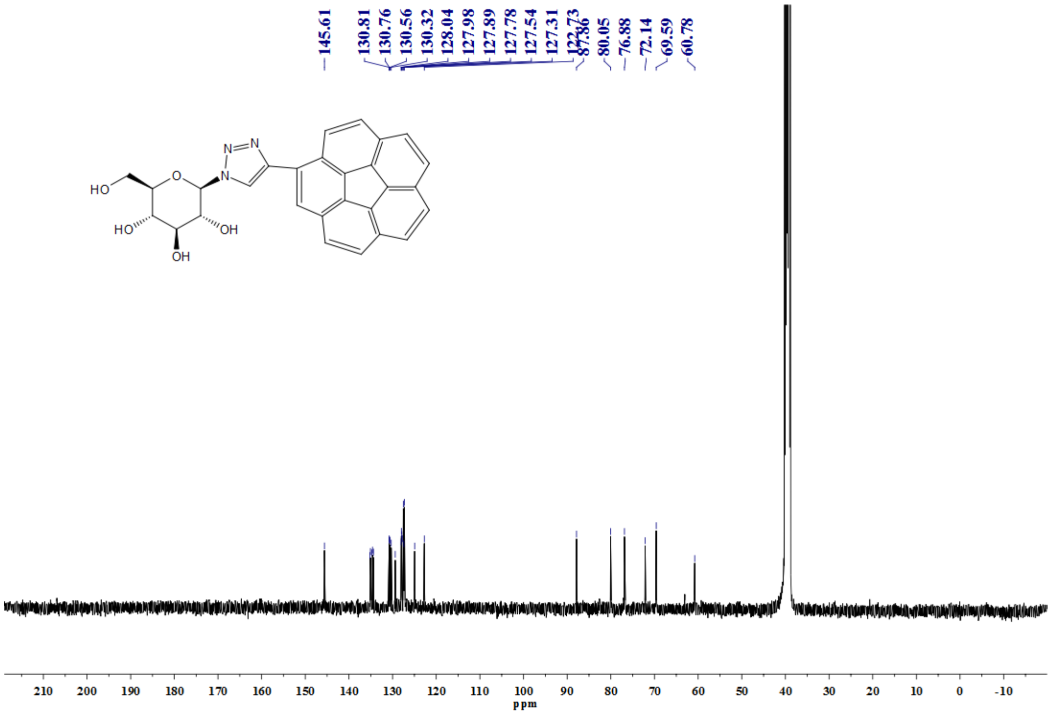

Fig. S2. ^{13}C NMR (100 MHz) spectrum of **Cor-glu** in DMSO-*d6*.

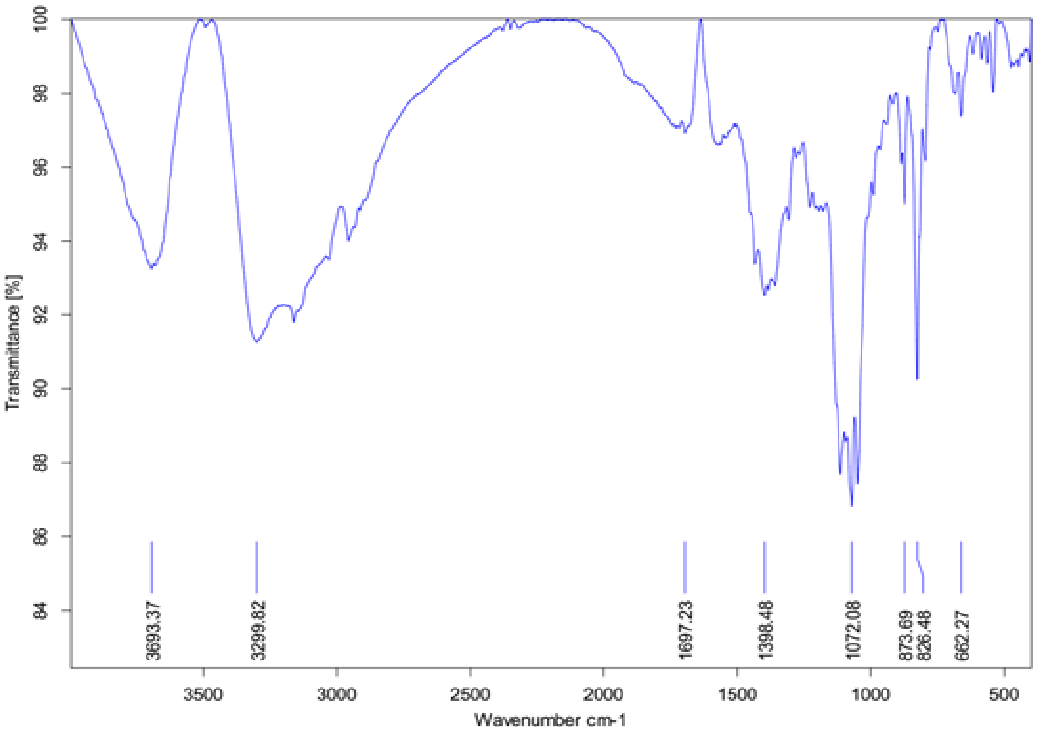

Fig. S3. IR (KBr) spectrum of **Cor-glu**.

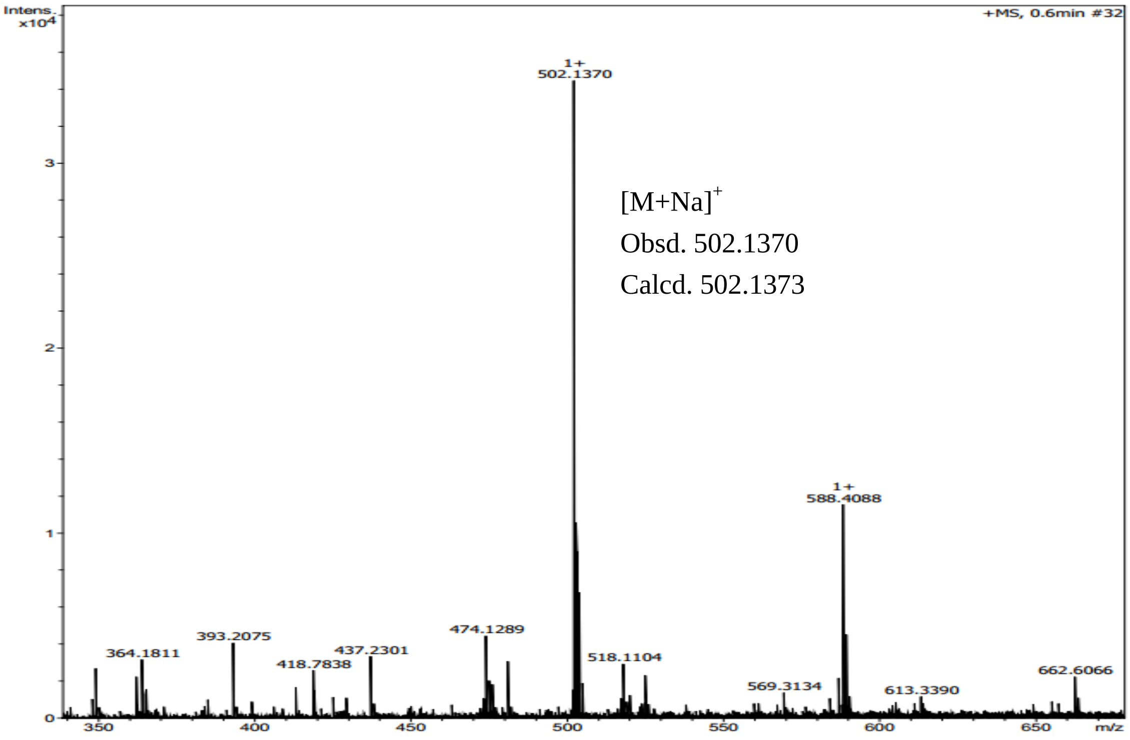

Fig. S4. ESI-MS spectrum of **Cor-glu**.

[Conditions] Column: 4.6 X 250 mm, Cosmosil PAQ-C18, 5 um
Eluent: 85% MeOH - 15% H2O
Rate: 1.0 ml/min, 254nm

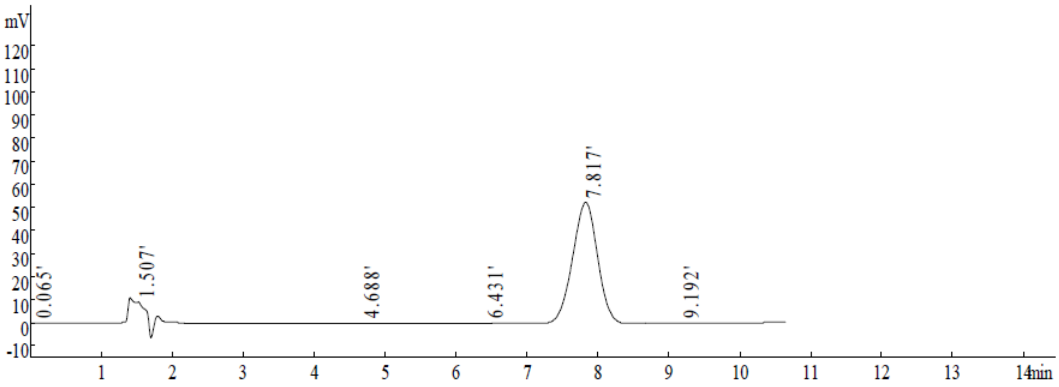

No.	RT (min)	Con.	Area	Peak width at half height
1	0.065	0.002874	39	2.426
2	1.507	3.841	51793	21.929
3	4.688	0.2085	2812	15.352
4	6.431	0.02361	318	9.343
5	7.817	95.25	1284341	22.989
6	9.192	0.6735	9081	40.804

Fig. S5. Purity analysis of **Cor-glu**.

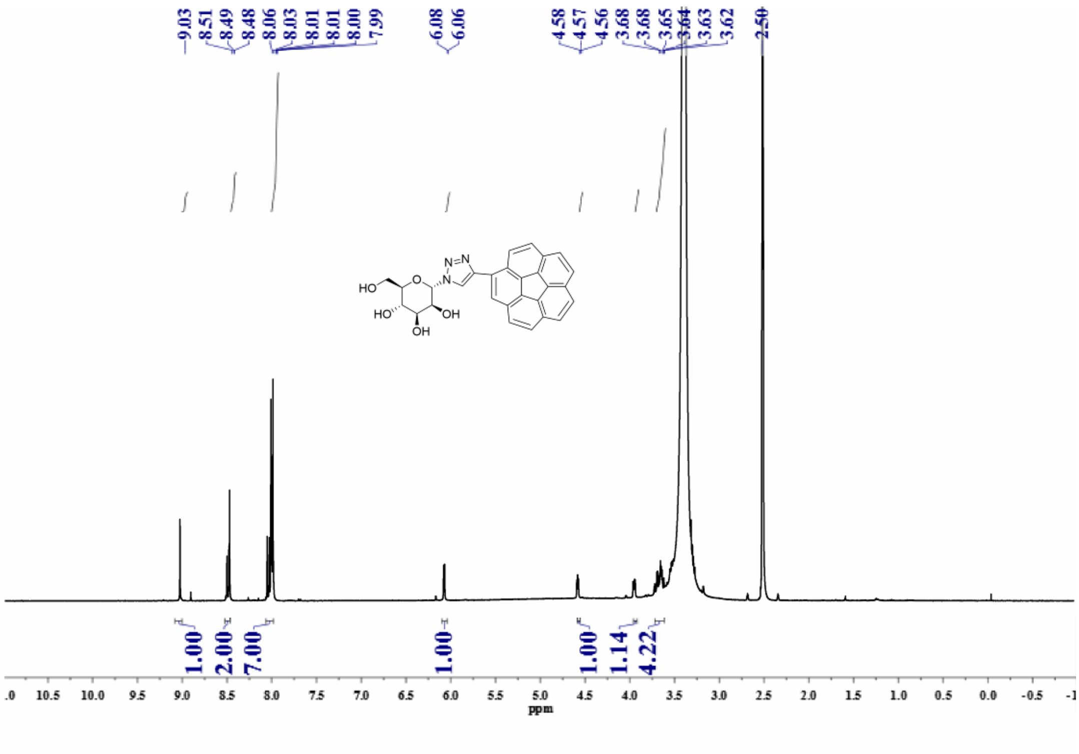

Fig. S6. ^{1}H NMR (400 MHz) spectrum of **Cor-man** in DMSO-*d6*.

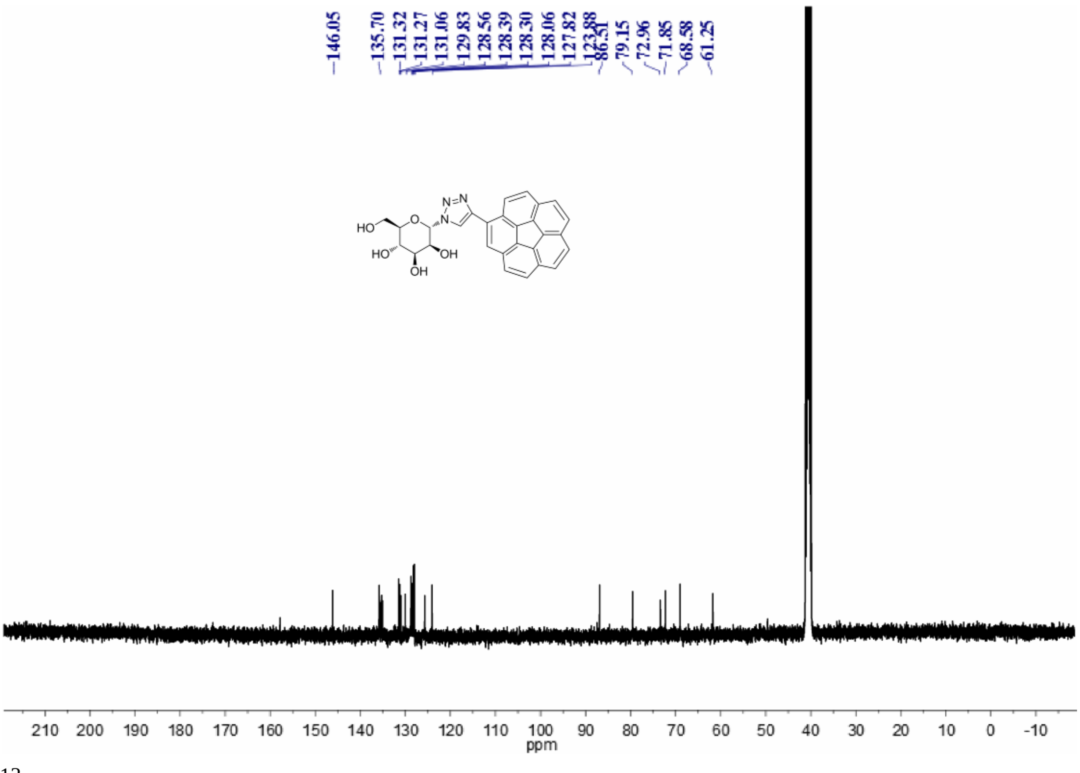

Fig. S7. ^{13}C NMR (100 MHz) spectrum of **Cor-man** in DMSO-*d6*.

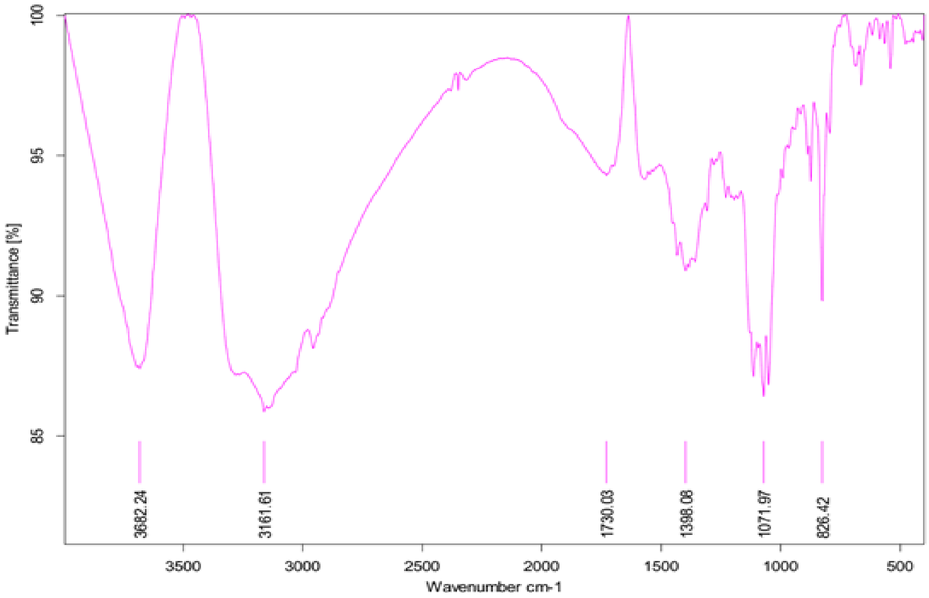

Fig. S8. IR (KBr) spectrum of **Cor-man**.

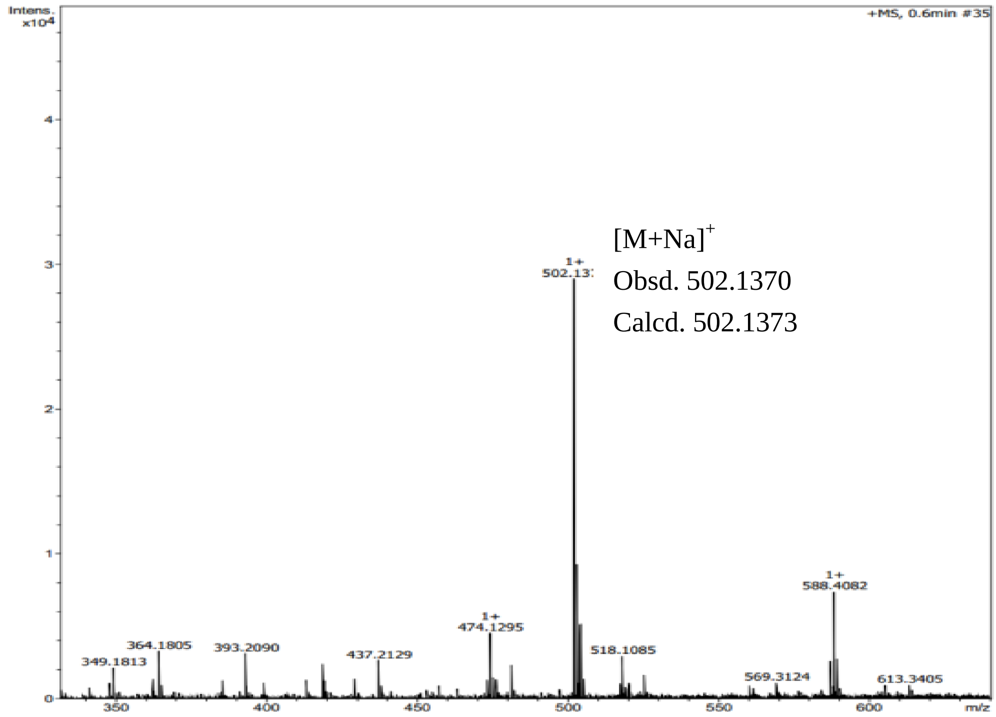

Fig. S9. ESI-MS spectrum of **Cor-man**.

[Conditions] Column: 4.6 X 250 mm, Cosmosil PAQ-C18, 5 um
Eluent: 85% MeOH - 15%H2O
Rate: 1.0 ml/min, 254 nm

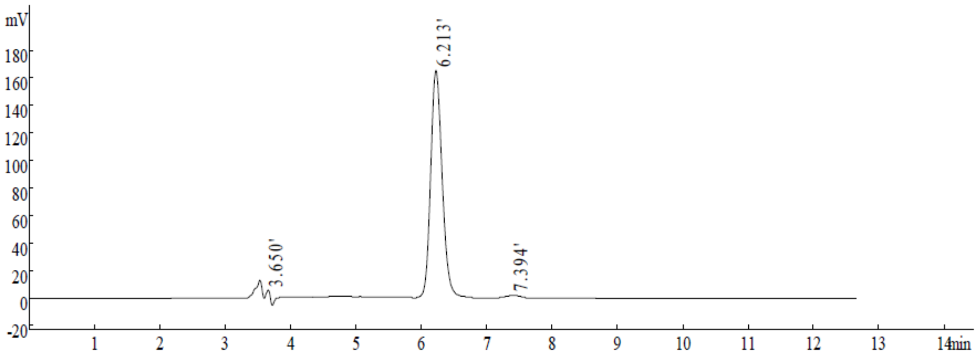

No.	RT (min)	Con.	Area	Peak width at half height
1	0.021	0.006359	133	2.556
2	3.650	1.701	35678	3.724
3	6.213	96.91	2032150	11.561
4	7.394	1.377	28866	13.657

Fig. S10. Purity analysis of **Cor-man**.

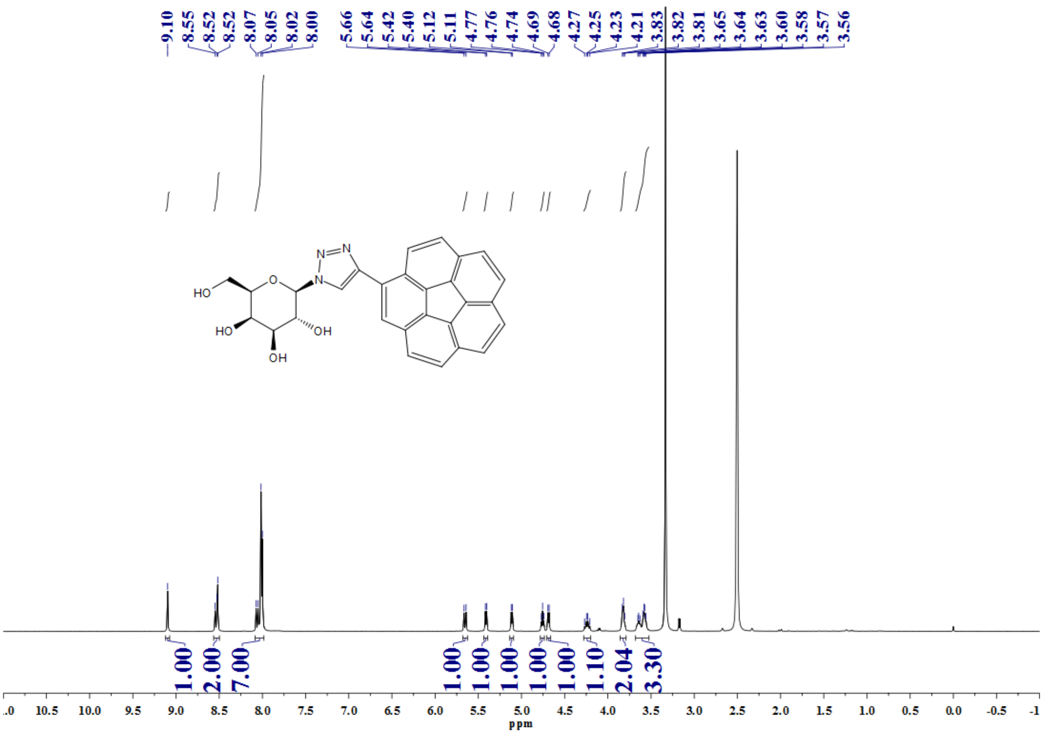

Fig. S11. ^{1}H NMR (400 MHz) spectrum of **Cor-gal** in DMSO-*d6*.

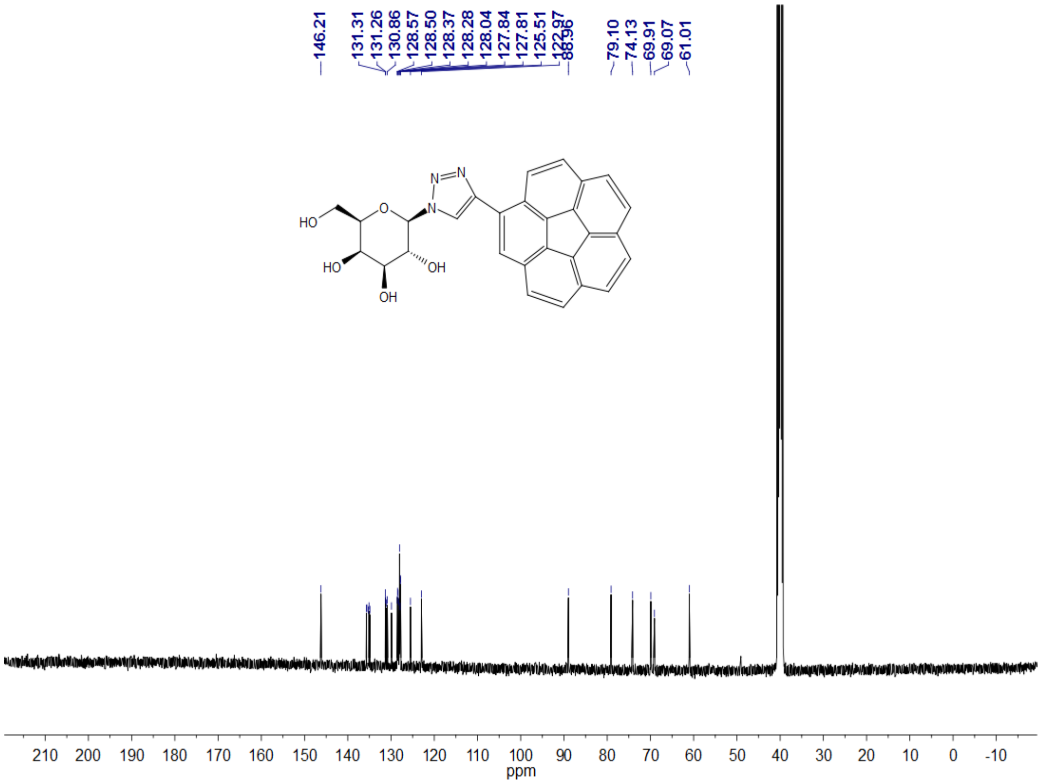

Fig. S12. ^{13}C NMR (100 MHz) spectrum of **Cor-gal** in DMSO-*d6*.

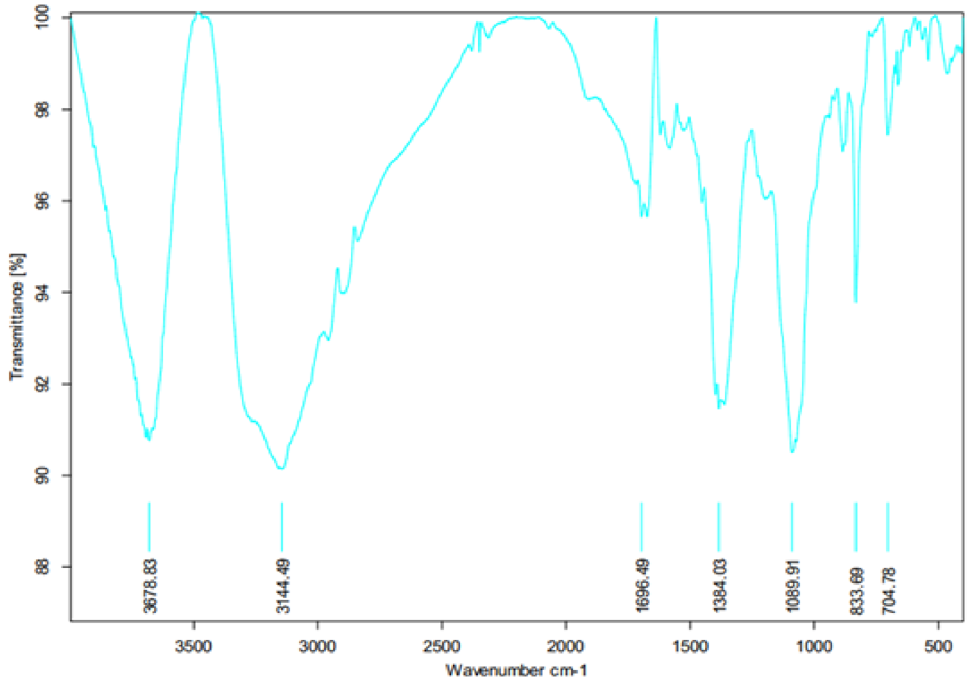

Fig. S13. IR (KBr) spectrum of **Cor-gal**.

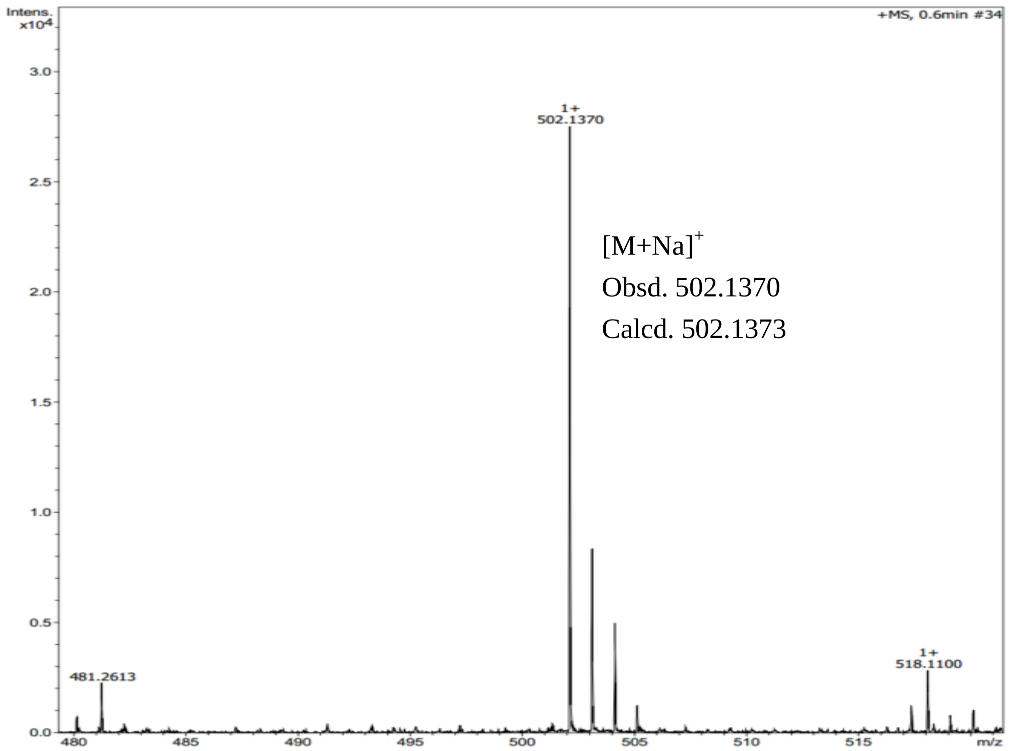

Fig. S14. ESI-MS spectrum of **Cor-gal**.

[Conditions] 4.6 X 250 mm, Cosmosil PAQ-C18, 5 um
Eluent: 85% MeOH - 15%H2O
Rate: 1.0 ml/min, 254 nm

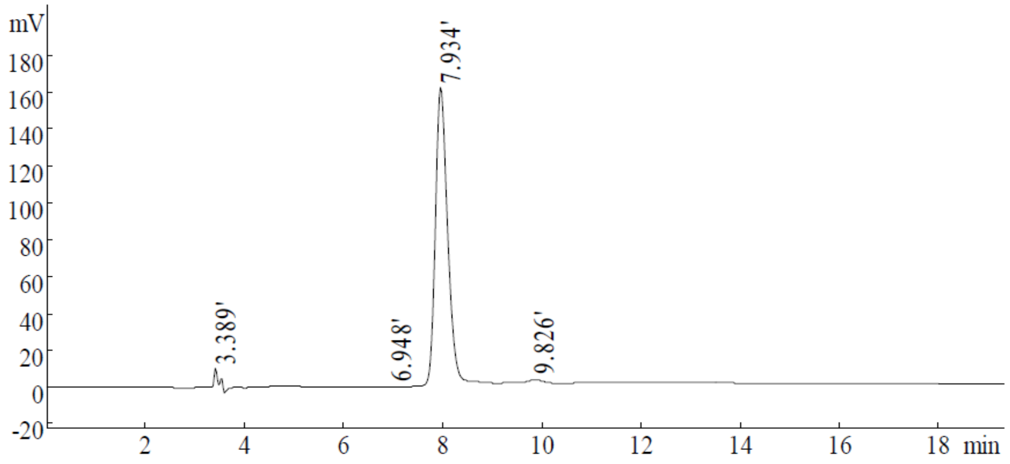

No.	RT(min)	Con.	Area	Peak width at half height
1	3.389	1.246	36633	3.214
2	6.948	0.5759	16927	30.107
3	7.934	96.36	2832031	16.363
4	9.826	1.812	53264	23.934

Fig. S15. Purity analysis of **Cor-gal**.

2. Fluorescence property

The fluorescent signal was measured at specific times (10~180 min) by using a Varioskan LUX multimode microplate reader (Thermo Fisher Scientific) to acquire the excitation and emission spectra of the corannulene-sugar conjugates. First, 1 mM stock solutions of corannulene and the conjugates in Acetonitrile or DMSO (Tokyo Chemical Industry) were prepared by vortexing followed by ultrasonication for 15 min. The stock solutions were further diluted with acetonitrile or PBS (pH 7.2) to 20 μM working solutions. A 96-well black polystyrene microplate (Thermo Fisher) was used for fluorescence measurements in the ranges of λex = 200–450 and λem = 368–600 nm.

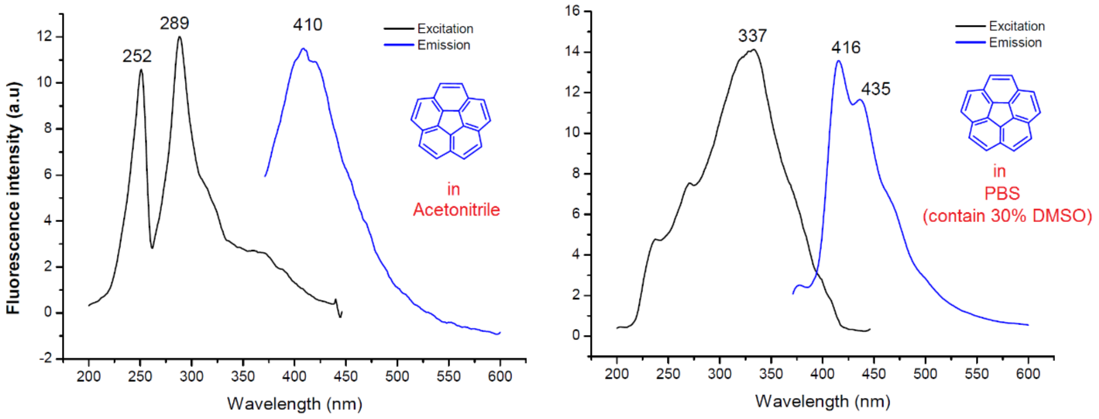

Fig. S16. Excitation and emission spectra of **corannulene** in different solvents.

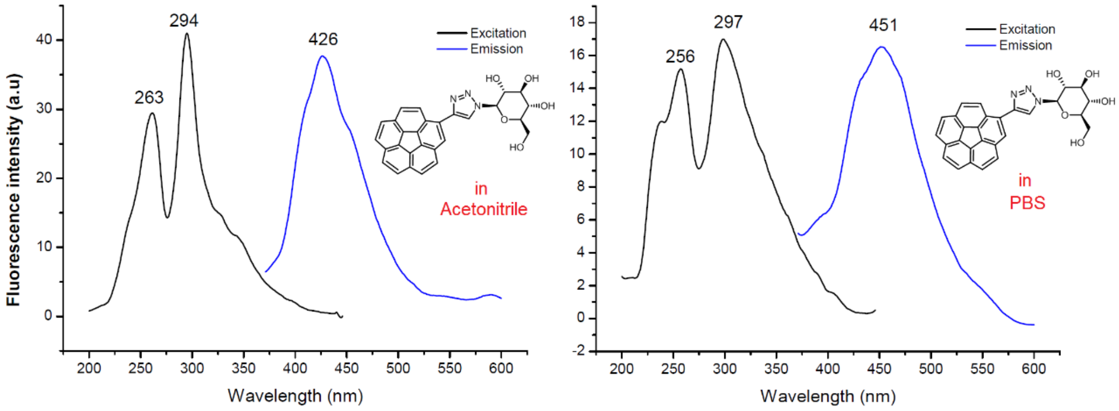

Fig. S17. Excitation and emission spectra of **Cor-glu** in different solvents.

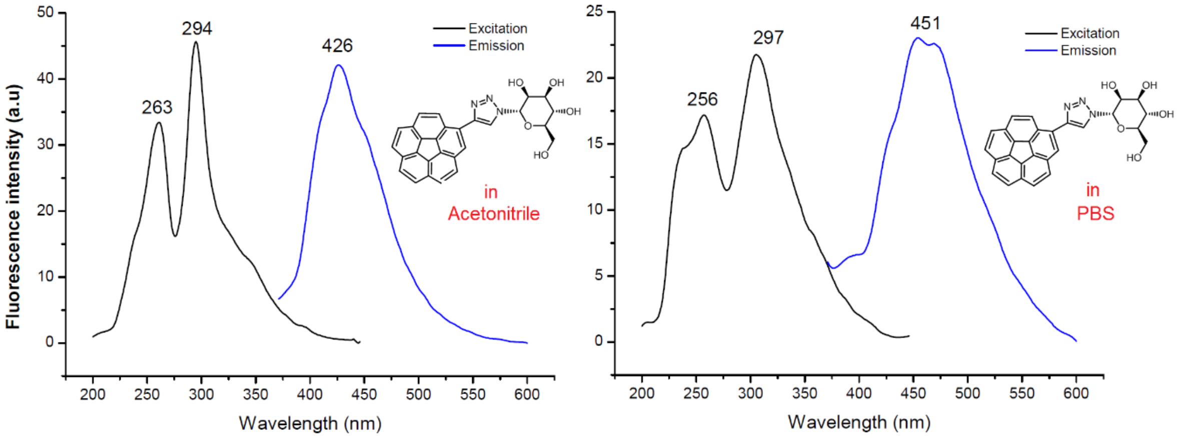

Fig. S18. Excitation and emission spectra of **Cor-man** in different solvents.

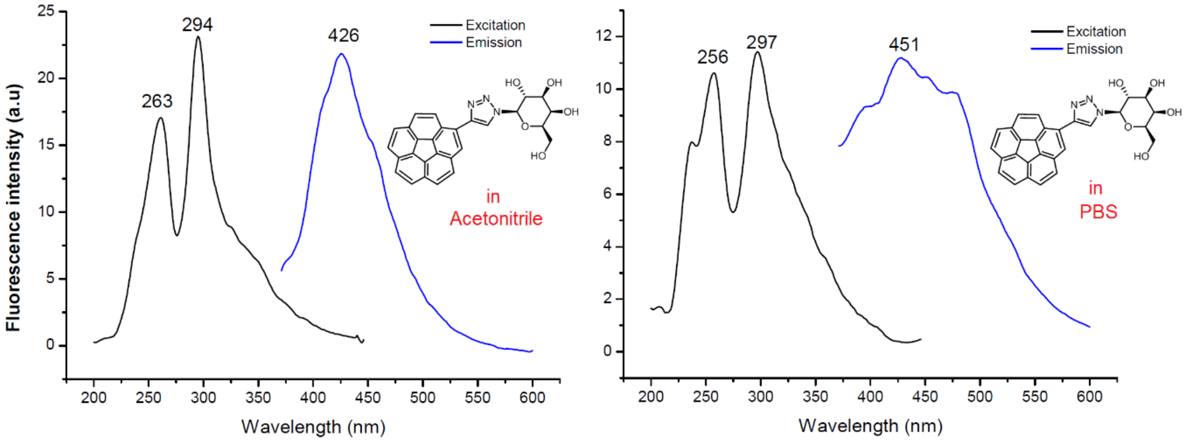

Fig. S19. Excitation and emission spectra of **Cor-gal** in different solvents.

3. *In Vitro* anticancer activity

Table S1. Cytotoxicity of the corannulene-sugar conjugates against different cancer cell lines.*

Compounds	IC50 (μM)								
	A549	H460	DU145	HepG2	HGC27	H929	Hela	HT29	BEAS-2B
Cor-glu	16.78 ± 2.13	28.09 ± 2.01	25.25 ± 2.01	20.00 ± 2.01	49.91 ± 2.00	21.07 ± 2.24	42.46 ± 2.98	46.95 ± 4.01	>100
Cor-man	3.47 ± 0.44	4.06 ± 0.37	3.13 ± 0.25	15.87 ± 1.88	4.30 ± 0.81	10.78 ± 0.89	38.26 ± 2.00	35.34 ± 3.01	>100
Cor-gal	6.02 ± 1.01	5.39 ± 0.41	4.21 ± 0.32	15.69 ± 2.41	40.49 ± 3.34	23.29 ± 1.01	31.75 ± 2.07	43.47 ± 2.99	>100
CPT	95.26 ± 6.03	24.09 ± 2.79	135.00 ± 5.11	36.88 ± 2.76	68.31 ± 4.88	32.40 ± 3.18	34.13 ± 3.01	115.25 ± 6.55	150± 7.23

*IC_{50} values were evaluated bt MTT assay over 72h. All compounds were tested in the same batch, and data are the mean ± SD of experiments performed three times in five replicates. CPT: carboplatin, A549: human lung carcinoma, H460: human lung cancer, DU145: human prostate cancer, HepG2: human liver cancer, HGC27: human gastric cancer, H929: human multiple myeloma, Hela: human cervical cancer, HT29: human colon cancer.

Table S2. Comparison of the cytotoxicities between Cor-sugars, cisplatin, carboplatin and DOX in A549 cancer cells.

Compounds	IC50 (μM)					
	CDDP	CPT	Cor-glu	Cor-man	Cor-gal	DOX
A549	7.18 ± 1.03	95.26 ± 6.03	16.78 ± 2.13	3.47 ± 0.44	6.02 ± 1.01	2.78 ± 0.35

4. GLUT-dependent cell uptake

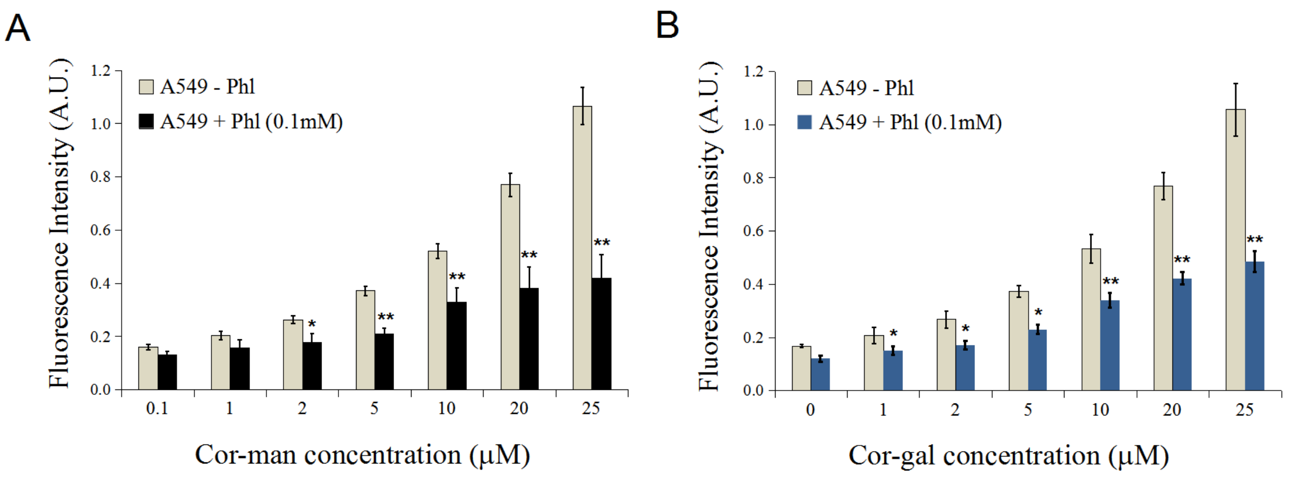

Fig. S20. Competitive inhibition assay of Cor-man and Cor-gal uptake in A549 cells using GLUT

inhibitor phloretin. a) Cor-man uptake with and without pre-treatment of GLUT inhibitor. b) Cor-gal uptake with and without pre-treatment of GLUT inhibitor. **P < 0.01, *P < 0.05 for 0.1mM phloretin treated cell uptake vs inhibitor non-treated groups.

5. Subcellular distribution of Cor-gal in A549 cells

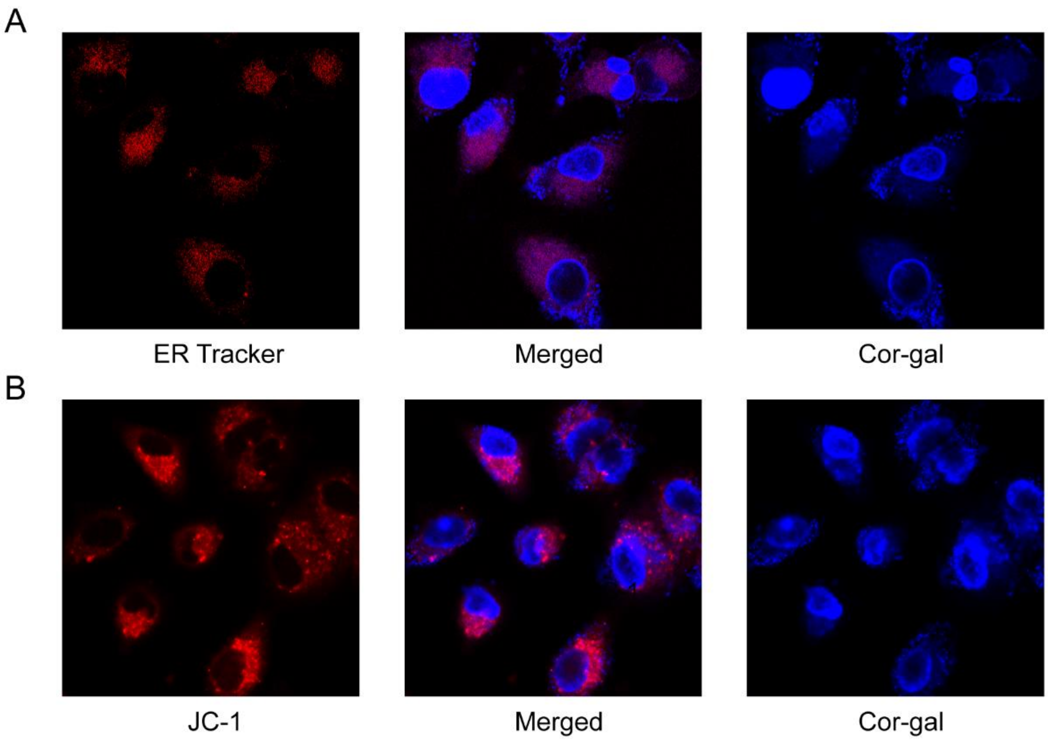

Fig. S21. Distribution of Cor-gal in subcellular organelles of A549 tumor cells. a) Distribution of Cor-gal in the endoplasmic reticulum by ER-Tracker Red staining and Cor-gal treatment. b) Assessment of mitochondrial distribution of Cor-gal with JC-1 staining and Cor-gal treatment. A549 cells were plated at a density of 2×10^5 cells/mL in a 12 mm Nunc glass bottom plate. Cells were incubated with corresponding staining dyes (ER Tracker: 1 μM, JC-1 probe: 10 μg/mL) by following the protocol provided by the manufacturer, and treated with 50 nM of Cor-gal at 37 °C for 30 min.

6. DNA interaction/circular dichroism

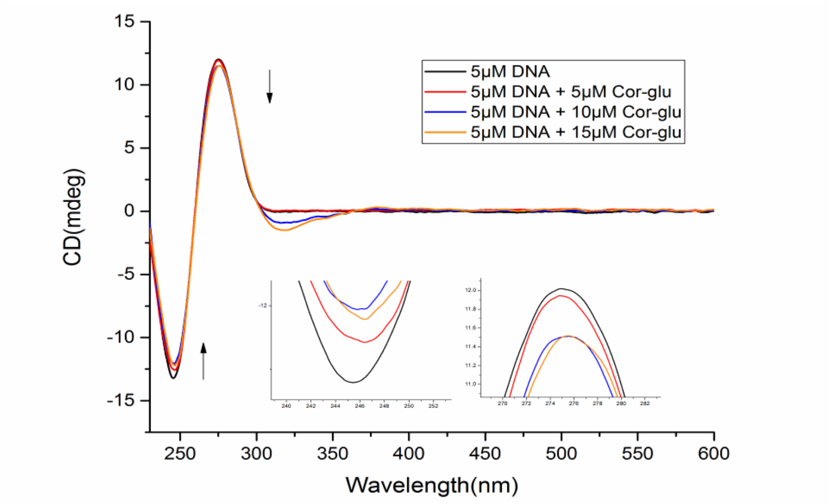

Fig. S22. CD spectra of hsDNA titrated by increasing ammount of **Cor-glu**. In Tris-HCl buffer (pH 7.2) at different molar ratio after equilibration at 25 °C.

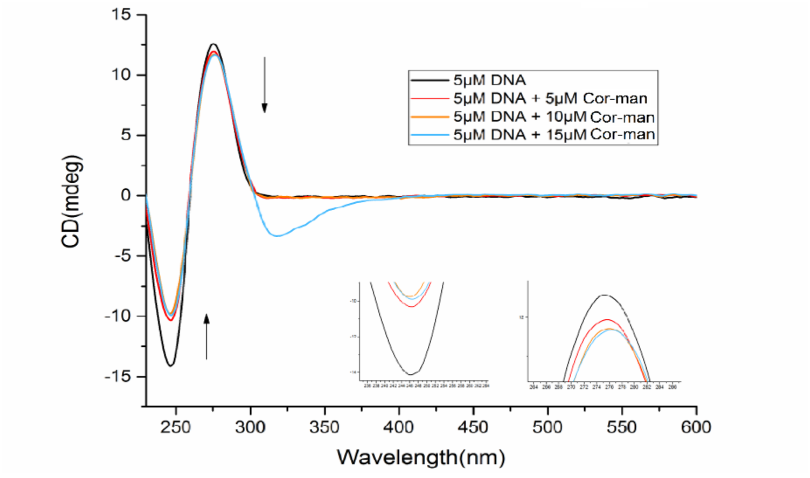

Fig. S23. CD spectra of hsDNA titrated by increasing ammount of **Cor-man**. In Tris-HCl buffer (pH 7.2) at different molar ratio after equilibration at 25 °C.

7. DNA-binding mechanism/ viscosity analyses

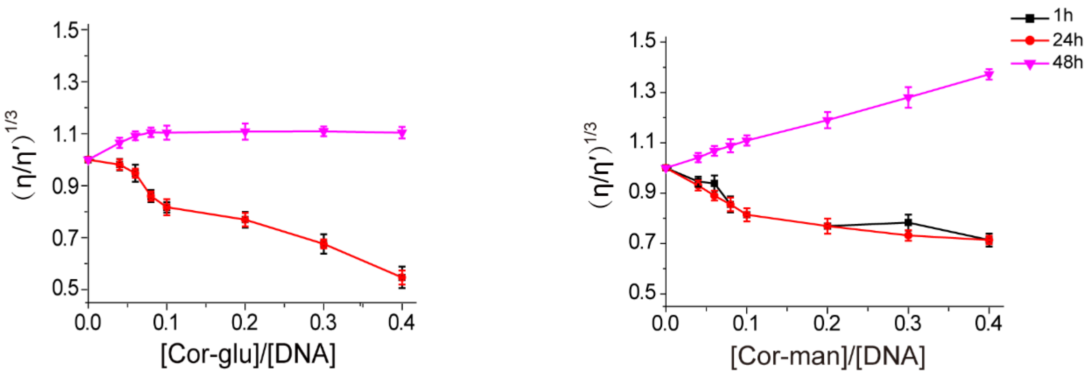

Fig. S24. Relative viscosity changes of hsDNA after treatment with Cor-glu and Cor-man for

different time periods (at 25 ± 0.1 °C, Tris-HCl pH = 7.2). The similar results were recorded for 1 h and 24 h incubated samples.

8. Stability assessment of Cor-sugars

Drug stability analysis. Cor-gal was selected for stability study. Cor-gal was dissolved in 100% DMSO to make 1 mM parent solution, and was then diluted with Tris-HCl buffer (pH 7.2) to 100 μM. Following the same procedure, 4 μM solution of benzylalcohol was prepared separately which was used as an internal standard. Cor-gal sample solution was stored at room temperature and the stability was measured after certain time period by using HPLC with benzyl alcohol as internal standard. 20 μL of the Cor-gal and 20 μL of the internal standard were mixed well together and 10 μL of the mixture was injected to the HPLC each time. [Cor-man in Tris-HCl buffer] and [Benzyl alcohol in Tris-HCl buffer] were measured under the same HPLC conditions as controls for retention times and for checking of buffer peaks.

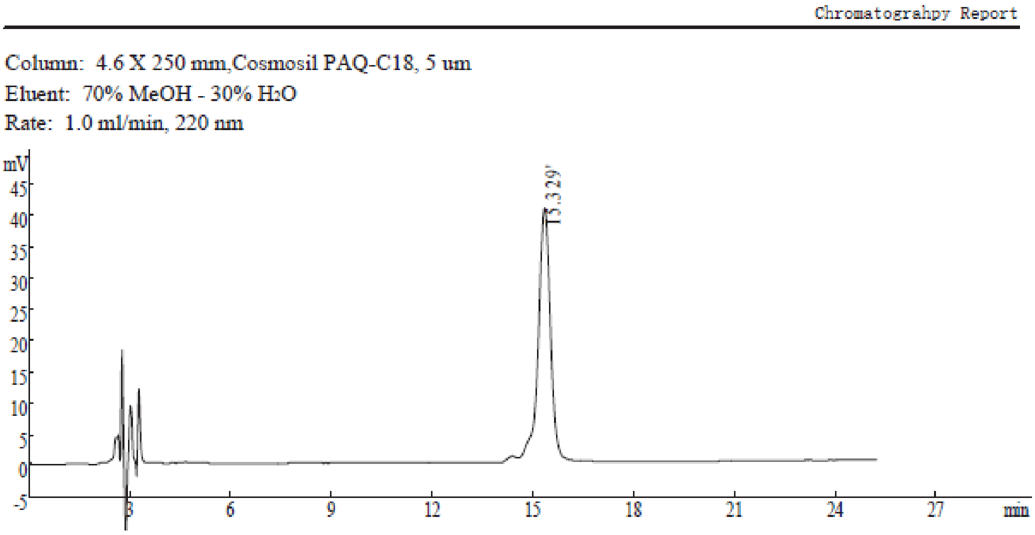

No.	RT(min)	Con.	Area	Peak width at half height
1	15.329	100	988968	23.142

Fig. S25. HPLC retention time check of [**Cor-gal** – Tris-HCl buffer] solution. Peak: Rt = 15.33: **Cor-gal**.

Chromatograhpy Report

Column: 4.6 X 250 mm,Cosmosil PAQ-C18, 5 um
Eluent: 70% MeOH - 30% H_2O
Rate: 1.0 ml/min, 220 nm

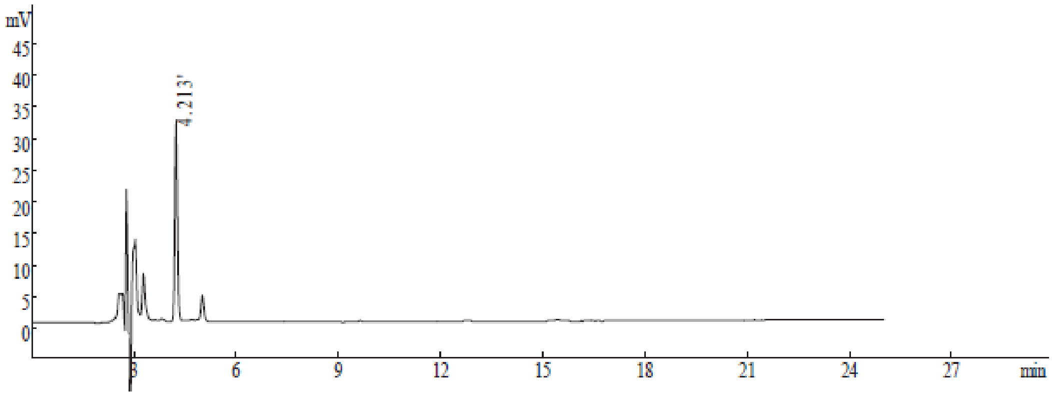

No.	RT(min)	Con.	Area	Peak width at half height
1	4.213	100	165165	4.743

Fig. S26. HPLC retention time check of [**Benzyl alcohol** – Tris-HCl buffer] solution. Peak: Rt = 4.21min: benzyl alcohol.

Chromatograhpy Report

Column: 4.6 X 250 mm,Cosmosil PAQ-C18, 5 um
Eluent: 70% MeOH - 30% H_2O
Rate: 1.0 ml/min, 220 nm

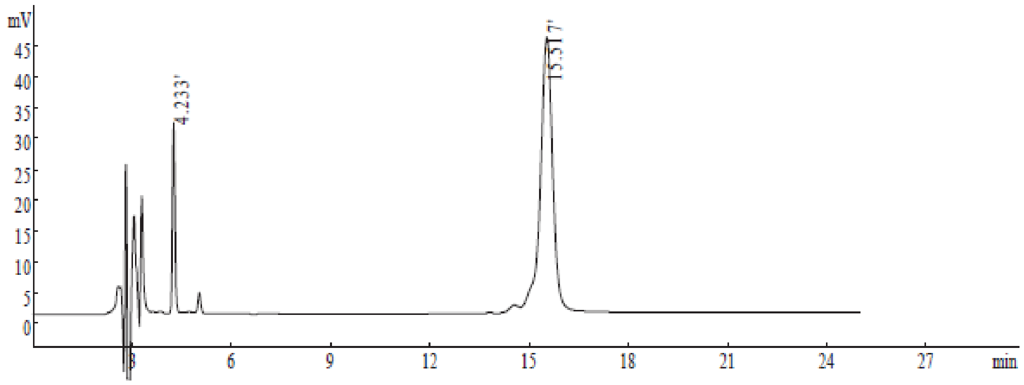

No.	RT(min)	Con.	Area	Peak width at half height
1	4.233	12.78	162623	4.723
2	15.517	87.22	1110155	23.714

Fig. S27. Stability analysis result of **Cor-gal** in Tris-HCl buffer (pH 7.2) at 0 day time point at r.t. Peaks: 1) Rt = 4.23 min: internal standard Benzyl Alcohol. 2) Rt = 15.5 min: **Cor-gal**.

Chromatograhpy Report

Column: 4.6 X 250 mm,Cosmosil PAQ-C18, 5 um
Eluent: 70% MeOH - 30% H_2O
Rate: 1.0 ml/min, 220 nm

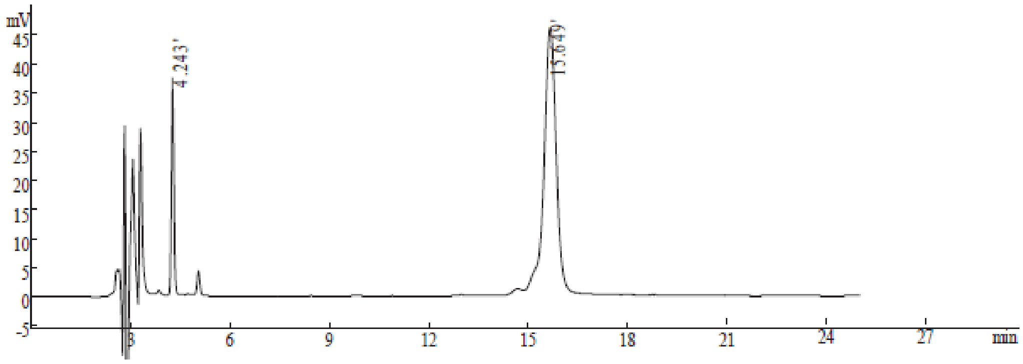

No.	RT(min)	Con.	Area	Peak width at half height
1	4.243	14.41	192790	4.739
2	15.649	85.59	1145431	23.698

Fig. S28. Stability analysis result of **Cor-gal** in Tris-HCl buffer (pH 7.2) after 5 days at r.t. Peaks: 1) Rt = 4.24 min: internal standard Benzyl Alcohol. 2) Rt = 15.6 min: Cor-man.

9. MD simulation study

Molecular docking study. To investigate the interactions between our sugar-conjugated compounds and DNA molecules, a 15-mer DNA structure in PDB databank were selected and used as template models. Cocrystallized ligands were extracted and DFT optimized Cor-gal was replaced respectively with the bound native drugs. For protein preparation, all hydrogen atoms were added randomly, and side chains were optimized during the receptor preparation. In the docking process, the receptor was kept rigid and the optimized ligands were set to be flexible during docking.

Molecular dynamics simulations. After docking process completed, all systems were subjected to the MD simulation study. All MD simulations were performed using YASARA program. Firstly, the values of the nucleic acids' pK_a shifts were predicted, the protonation states were assigned according to pH = 7.0, and the simulation cell was filled with 0.9% NaCl. Prior to MD simulations, energy minimizations including steepest descent minimization and simulated annealing minimization were performed in solvated condition. After minimization, the production simulation of 200 ns was then performed with the smooth particle-mesh Ewald (PME) method. The all-atom AMBER14 force field was used in our simulation. The value of non-bonded cutoff was set to 8.0 Å, the integration time step was set to 4 fs, and the constrained bonds were formed by the hydrogen atoms. The NPT ensemble (pressure P, temperature T, and number of atoms N) with the constant pressure of 1 atm and temperature of 298 K were used in this study. Molecular graphics and analysis of the simulation results were performed with the UCSF Chimera package. The binding energy was obtained by calculating the energy at infinite distance (between the selected object and the rest of the soup, i.e., the unbound state) and subtracting the energy of the soup (i.e., the bound state). The more positive the binding energy, the more favorable the interaction in the context would be expected. Here, the binding energies (i.e., the energy required to disassemble a whole into separate parts) were calculated

by mean of boundary elements theory.

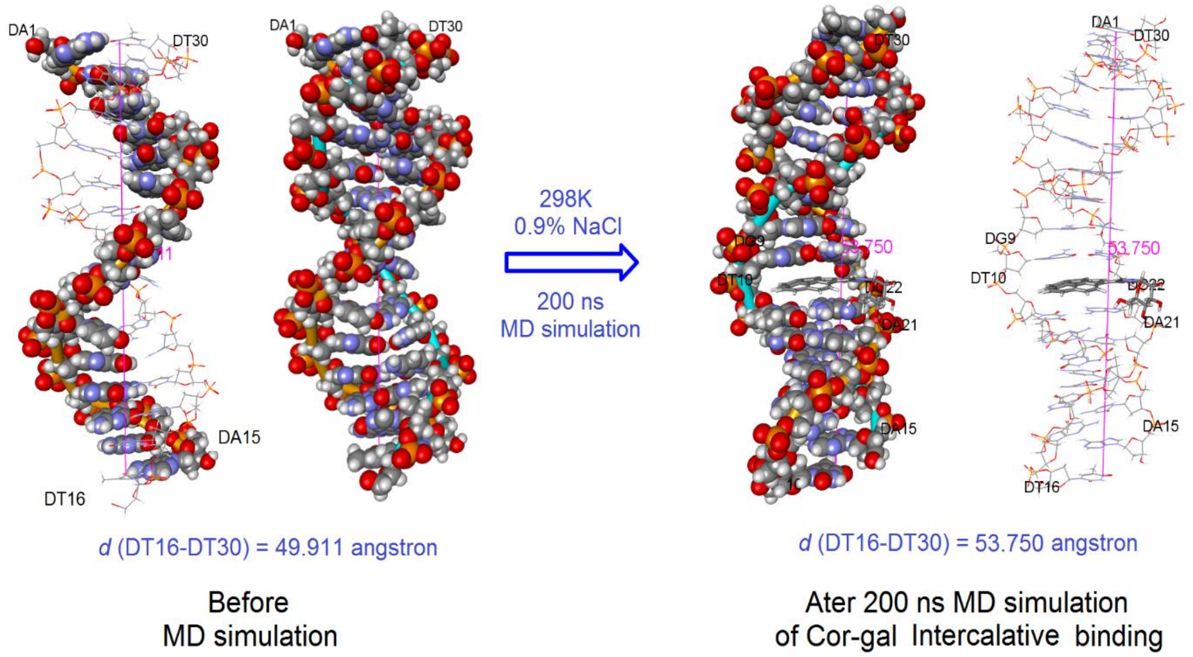

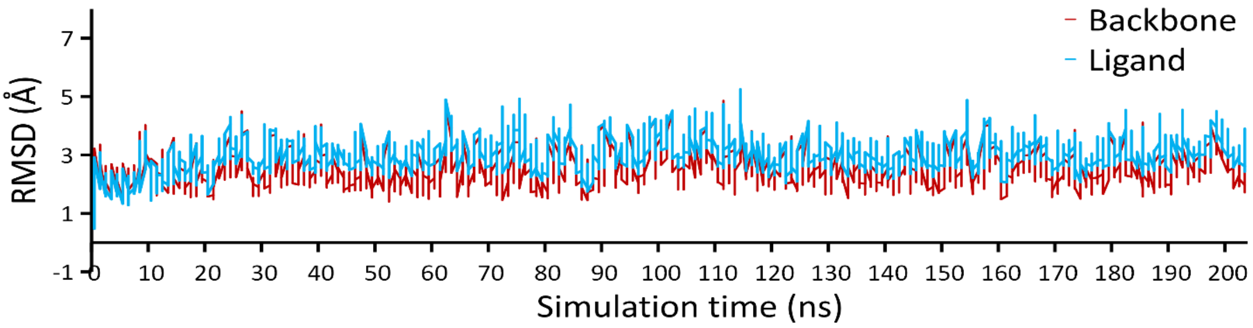

Fig. S29. Intercalative binding of Cor-gal/DNA. Upper) 200 ns MD simulation results of intercalative binding of Cor-gal. Bottom) Root mean square deviation (RMSD) fluctuations of 200 ns MD simulations for **Cor-gal**/DNA. RMSD reflect the backbone atoms compared with the initial structures.

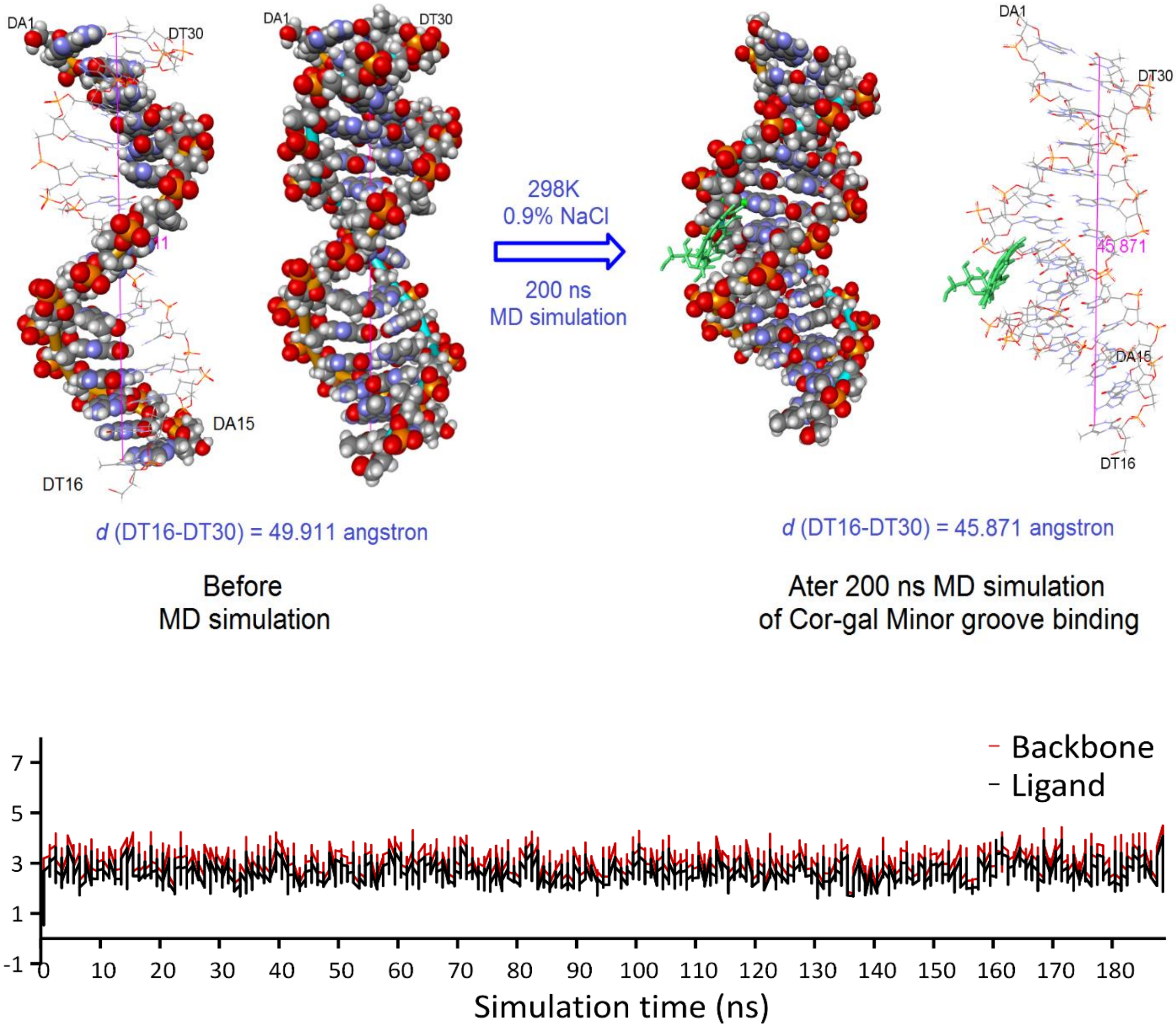

Fig. S30. Minor groove binding of Cor-gal/DNA. Upper) 200 ns MD simulation results on Cor-gal binding mode in minor groove. Bottom) Root mean square deviation (RMSD) fluctuations of 200 ns MD simulations for **Cor-gal**/DNA. RMSD reflect the backbone atoms compared with the initial structures.

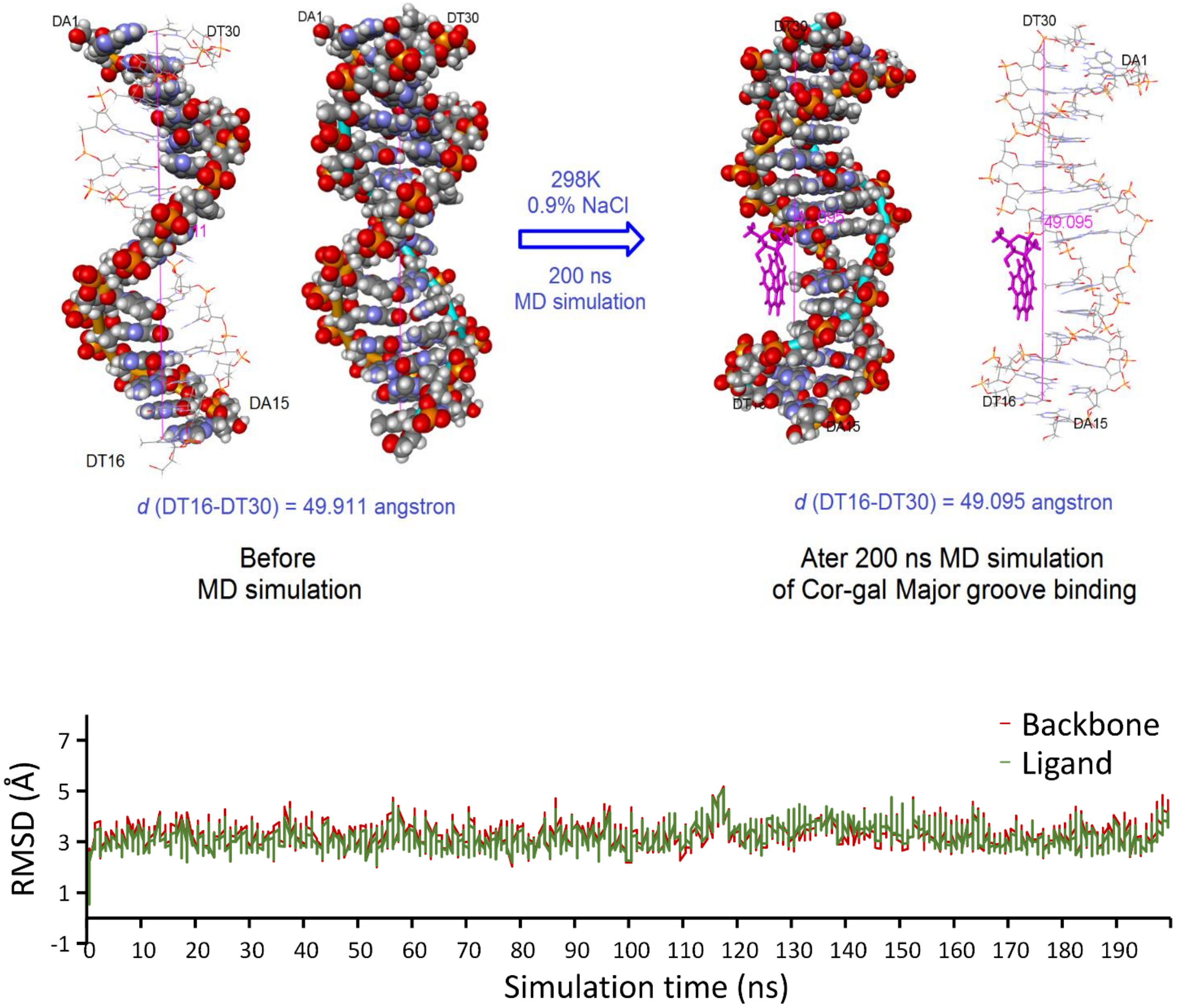

Fig. S31. Major groove binding of Cor-gal/DNA. Upper) 200 ns MD simulation results on Cor-gal binding mode in major groove. Bottom) Root mean square deviation (RMSD) fluctuations of 200 ns MD simulations for **Cor-gal**/DNA. RMSD reflect the backbone atoms compared with the initial structures.

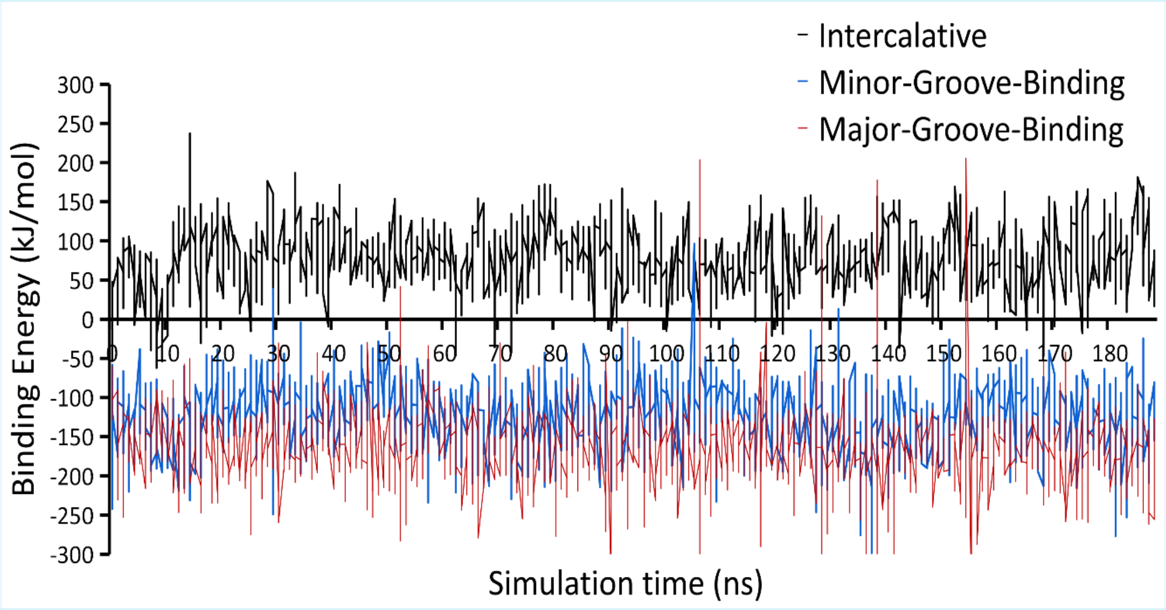

Fig. S32. Binding energy of Cor-gal with 15-mer DNA (2MG8) calculated by boundary elements theory. More positive binding energies indicate more favorable binding of the compound with the DNA. Intercalation of **Cor-gal** with DNA exhibited the most stable interaction followed by minor groove binding and major groove binding.

Table S3. Summary of the statistical results for binding energies by methods of boundary elements obtained from the 200 ns MD simulation study of **Cor-man**/DNA in different interaction modes*.

	Binding Energy (kJ/mol) (by boundary elements theory)		
	Intercalation	Minor groove binding	Major groove binding
**$\bar{E}$ (kJ/mol)**	**76.588**	**-122.918**	**-161.757**

* More positive binding energies indicate more favorable binding of the compound with the DNA. $\bar{E}$: Average binding energy over 200 ns MD simulation process.

10. *In vivo* toxicity in zebrafish

Zebrafish care and handling

AB strain zebrafish were used for experiments in this study. Zebrafish embryos were acquired by a natural pair-wise mating method. After fertilization, the embryos were incubated and maintained at 35 °C in fish water (0.2% Instant Ocean salt in deionized water, pH 6.9–7.2, conductivity of 480-510 mS/cm, and hardness of 53.7-71.6 mg/L with $CaCO_3$). Larvae at 2 days after fertilization (dpf) were collected and used for maximum tolerated dose (MTD) and drug efficacy studies. A total of 30 zebrafish larvae were used in each experimental group, and the compounds were administered via intravenous injection by a microinjection method. The treated larvae were assessed at 48 h following drug administration. The larvae were anaesthetized in 0.016% (w/v) tricaine prior to observation. Tumour cell analysis was performed by calculating their fluorescence intensity using Nikon NIS-Elements D 3.10 Advanced image processing software after fluorescence microscopy imaging (Nikon, AZ100). The University Animal Care and Use Committee approved the animal experimental

procedures described in this study. These procedures are consistent with the guidelines of the American Veterinary Medical Association Panel on Euthanasia.

Tumor cell labelling and transgenic zebrafish model

Before transplantation of the cancer cells to zebrafish, human non-small cell lung cancer A549 cells were labelled using the red fluorescent dye CM-Dil. The A549 cells were incubated with CM-Dil for 5 min at 37 °C and then kept at 4 °C for 15 min. After centrifugation, the cells were rinsed with cold PBS and trypsinized before microinjection (dissecting microscope: SZX7, OLYMPUS, Japan; microinjector: IM300, NARISHIGE, Japan). The microneedle was attached to an air-driven cell tram. A total of 200 CM-DiI-labelled A549 cells in 15 nL PBS were microinjected into 2 dpf larvae through the perivitelline space between the yolk and the periderm. The larvae were kept at 33 °C in Holt buffer solution (60 mM NaCl, 2.4 mM $NaHCO_3$, 0.9 mM $CaCl_2$, 0.67 mM KCl) for 24 h.

MTD determination and safety assessment

The MTD was evaluated for all drugs with zebrafish larvae at 3 dpf. Each experimental group contains 30 larvae. The larvae were anaesthetized in 0.016% (w/v) tricaine, and drugs (DOX, Cor-glu, Cor-man and Cor-gal) at the desired concentration (0.1-20 ng/fish for DOX, 2.2-40 ng/fish for Cor-sugars) were microinjected intravenously (i.v.) into each larva in the testing groups (2 nL per fish). Embryos were cultured in 6-well plates at 30 embryos per well for 2 days. The PBS-treated group and PBS containing 0.5% DMSO-treated group were used as two control groups. The embryos were visually analysed using a stereomicroscope (AZ100， Nikon， Japan) for organ-specific toxicity. Abnormal heart rate, arrhythmia, abnormal circulation, pericardial edema and abnormal heart chamber morphology were used to evaluate the standard of cardiac toxicity; misshapen brain was analysed for CNS toxicity; liver size and colour were assessed for hepatotoxicity; and cyst formation and trunk edema were evaluated for renal toxicity.

Table S4. Summary of the drug safety and maximum tolerated concentration studies in zebrafish for doxorubicin and the corannulene-sugar conjugates.*

Groups	Dose(ng/fish)	Larveas/Group	Deaths	Mortality (%)	Toxicity Phenotype
Control-1(PBS)		30	0	0	Normal
Control-2(Solvent)		30	0	0	Normal
**DOX**	20	30	20	67	Severe renal and cardiac edema with heart and kidney failure
	15	30	3	10	30% cardiac and renal edema
	10	30	1	3.3	Normal
	5	30	0	0	Normal
	2.5	30	0	0	Normal
	1	30	0	0	Normal
	0.5	30	0	0	Normal

	0.1	30	0	0	Normal
**Cor-glu**	40	30	1	3.3	Normal
	30	30	0	0	Normal
	20	30	0	0	Normal
	13.3	30	0	0	Normal
	6.7	30	0	0	Normal
	4.4	30	0	0	Normal
	2.2	30	0	0	Normal
**Cor-man**	40	30	1	3.3	Normal
	30	30	0	0	Normal
	20	30	0	0	Normal
	13.3	30	0	0	Normal
	6.7	30	0	0	Normal
	4.4	30	0	0	Normal
	2.2	30	0	0	Normal
**Cor-gal**	40	30	1	3.3	Normal
	30	30	0	0	Normal
	20	30	0	0	Normal
	13.3	30	0	0	Normal
	6.7	30	0	0	Normal
	4.4	30	0	0	Normal
	2.2	30	0	0	Normal

*Larveas of 3 days after fertilization (dpf) were used for maximum tolerated dose (MTD) studie. 30 zebrafish larveas were used in each experimental group and the drugs were administered via intravenous injection by a microinjection method. The treated larbeas were assessed at 48 hours following the drug administration.

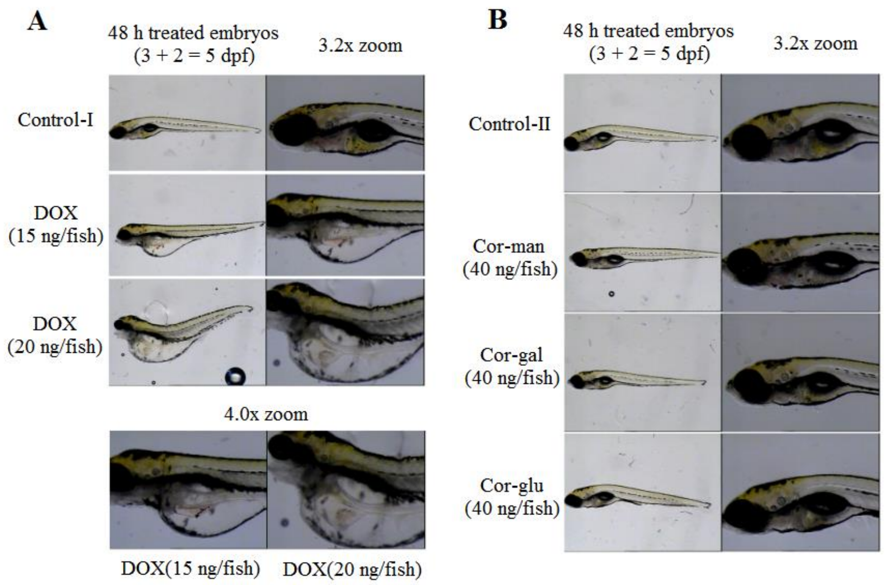

Fig. S33. A) Doxorubicin induced severe renal and pericardial edema under 15-20 ng/fish *i.v.*

treatment. B) No abnormal phenotypes detected up to 40 ng/fish *i.v.* treatment of the **Cor-sugars**. 3 dpf larvae was used for MTD study, each experimental group contain 30 larveas. The *i.v.* microinjected learveas were assessed at 48 hours following the drug administration. Control-1: PBS group. Control-2: PBS contains 0.5% of DMSO was used for solvent group.

11. *In vivo* fffficacy study in zebrafish xenograft

In vivo anticancer efficacy study

In vivo efficacy studies were performed at the drug screening platform of Hanzgou Hunter Biotechnology, Inc. A549 transgenic AB wild-type zebrafish larvae (transplanted at 2 dpf, incubated and maintained at 35 °C in fish water until 3 dpf) were distributed in 6-well plates with 30 larvae in each well. Then, the larvae were anaesthetized in 0.016% (w/v) tricaine, and drugs (DOX, Cor-glu, Cor-man and Cor-gal) at the desired doses (10 and 20 ng/fish for Cor-sugars, 10 ng/fish for DOX) were microinjected i.v. into each larva in the testing groups (2 nL per fish). The PBS-injected group was used as a control group. After the drugs were administered, the larvae were cultured at 35 °C for 2 days. At the end of the efficacy study, 10 larvae were randomly selected, anaesthetized with 0.016% (w/v) tricaine and imaged under a fluorescence microscope (AZ100, Nikon，Japan) equipped with a DP2-BSW digital camera (Olympus, Inc.). Tumour cell fluorescence images were taken with a fluorescence microscope, and fluorescence intensity was analysed using NIS-Elements D 3.10 software. Tumour inhibition rates (%) = (fluorescence intensity of control group-fluorescence intensity of drug treated group)/fluorescence intensity of control group × 100%. The animal experiments were performed in accordance with the approval from the Chinese Academy of Medical Sciences, and all studies were performed in accordance with the approved guidelines from the China Zebrafish Resource Center.

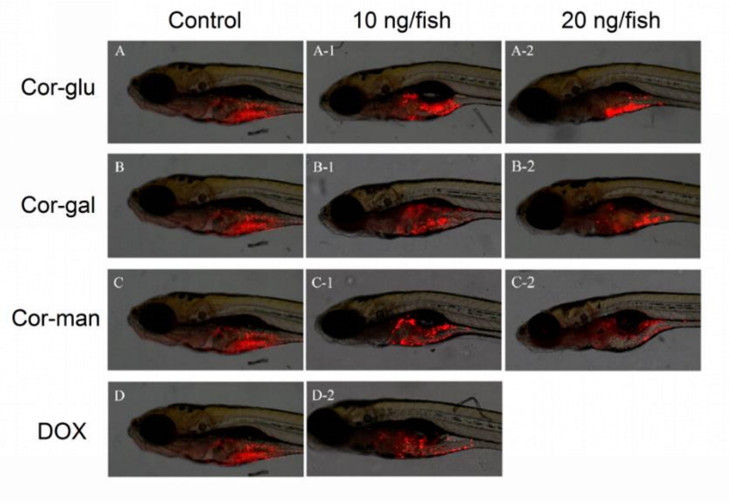

Fig. S34. Inhibition of the transplanted A549 tumor masses after a 2-day period of drug treatment.
